# Nanomechanical Photothermal Near Infrared Spectromicroscopy of Individual Nanorods

**DOI:** 10.1021/acsphotonics.3c00937

**Published:** 2023-09-20

**Authors:** Kostas Kanellopulos, Robert G. West, Silvan Schmid

**Affiliations:** Institute of Sensor and Actuator Systems, TU Wien, Gusshausstrasse 27-29, 1040 Vienna, Austria

**Keywords:** nanoabsorber, nanomechanical, photothermal, absorption, polarization, plasmon, spectroscopy

## Abstract

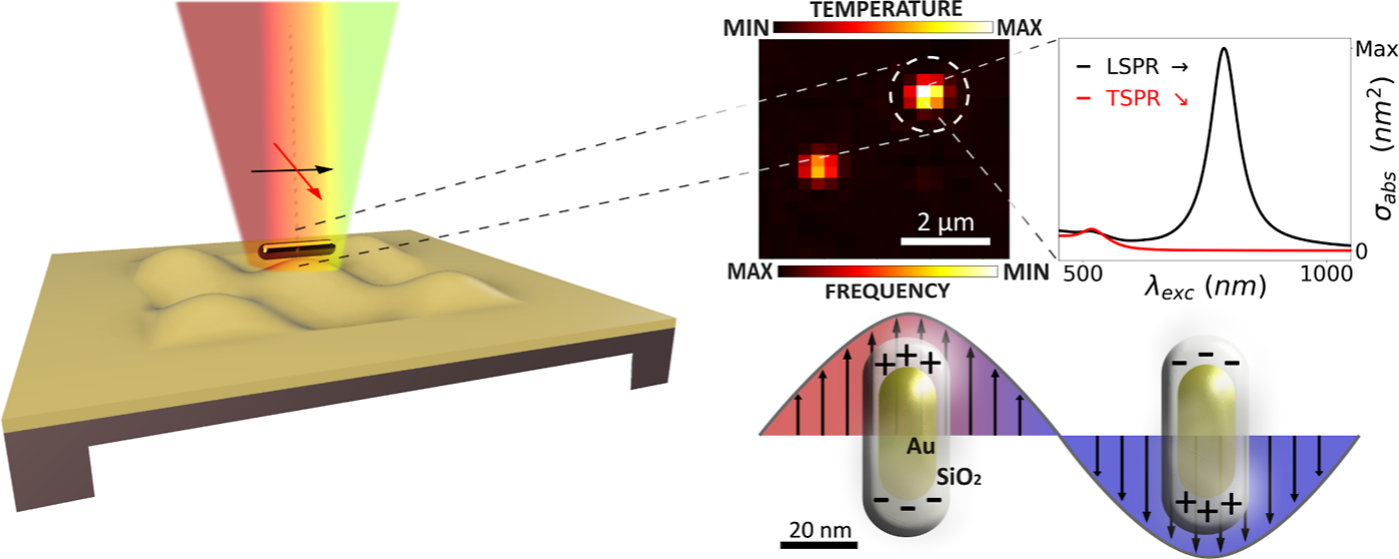

Understanding light-matter interaction at the nanoscale requires probing the optical properties of matter at the individual nanoabsorber level. To this end, we developed a nanomechanical photothermal sensing platform that can be used as a full spectromicroscopy tool for single molecule and single particle analysis. As a demonstration, the absorption cross-section of individual gold nanorods is resolved from a spectroscopic and polarization standpoint. By exploiting the capabilities of nanomechanical photothermal spectromicroscopy, the longitudinal localized surface plasmon resonance in the NIR range is unraveled and quantitatively characterized. The polarization features of the transversal surface plasmon resonance in the VIS range are also analyzed. The measurements are compared with the finite element method, elucidating the role played by electron surface and bulk scattering in these plasmonic nanostructures, as well as the interaction between the nanoabsorber and the nanoresonator, ultimately resulting in absorption strength modulation. Finally, a comprehensive comparison is conducted, evaluating the signal-to-noise ratio of nanomechanical photothermal spectroscopy against other cutting-edge single molecule and particle spectroscopy techniques. This analysis highlights the remarkable potential of nanomechanical photothermal spectroscopy due to its exceptional sensitivity.

## Introduction

The advent and development of optical single-molecule and single-particle measurement techniques have had a tremendous impact on scientific research for the past 30 years.^1^ The detection of single objects at the nanoscale level affords us a unique perspective on the interactions occurring between these tiny entities and their local environment, revealing their heterogeneity without relying on ensemble average information. Due to its high signal-to-noise ratio (SNR), optical fluorescence-based detection approaches have rapidly evolved and are, nowadays, routinely employed in a huge variety of scientific fields, from biology to condensed matter to the design and engineering of novel materials. However, the fluorescent label can photobleach or quench as well as alter the system under study. For this reason, the scientific community has pushed toward the development of label-free single-molecule detection schemes^2^ such as iSCAT,^3,4^ ground-state depletion microscopy,^5^ and photothermal microscopy,^6,7^ among others. All these approaches rely on the absorption rather than scattering of the nanoobject upon illumination of a probing light. The rationale behind this choice lies in the fact that, while the optical scattering cross-section scales quadratically with the target volume (σ_scatt_ ∝ *V*^2^), the optical absorption cross-section scales linearly with it (σ_abs_ ∝ *V*).^8,9^ In other words, the smaller the target size is, the more effectively its absorption properties can be interrogated. More specifically, this central aspect shows also the advantage of nanomechanical absorption spectroscopy over other fully optical single-molecule spectroscopic methods, such as surface-enhanced Raman scattering^10^ or tip-enhanced Raman spectroscopy.^11^ The former approach uses the strong near-field enhancement at the nanoscale on the surface of plasmonic nanoparticles or nanostructures (so-called hotspots) to increase the SNR of Stokes-shift Raman scattering. However, the plasmonic nanostructure fabrication and control of the placement of the particles/molecules in the sites of interest increase the overall complexity of the measurement procedure. The latter approach uses a nanoscopic probe to scan an area where the molecules of interest are fixed, requiring precise control over the tip fabrication process. In contrast, nanomechanical absorption spectroscopy overcomes all this complexity as it measures directly the nonradiative energy losses of the illuminated molecule, not limited to any specific sample preparation. In other words, the molecule itself becomes part of the detector due to the interplay between its absorption properties and the light excitation used. The reduced analyte-detector distance likewise results in a reduction of noise and unwanted external interference. Based on this consideration, it has been possible to detect and image single molecules by nanomechanical photothermal sensing.^7^ This work has been made possible by the previous research, which showed the ability of nanomechanical resonators to detect and quantify the absorption of single plasmonic^12–14^ and polymer^15^ nanoparticles via photothermal heating. It is worth noting that this photothermal spectromicroscopy approach is not based on the thermo-optical effect as in photothermal contrast microscopy,^6,16–19^ where the absorber is detected due to the temperature dependence of the surrounding embedding medium refractive index (glycerol, thermotropic liquid crystal, near-critical Xe or CO_2_)^6,20^ via modulation of the scattering of a second probing laser. In nanomechanical photothermal spectromicroscopy, this thermal effect consists instead of a stress reduction in the nanomechanical resonator, detuning its resonance frequency upon illumination of the nanoabsorber.

Here, this work pushes further the boundaries of single-molecule nanomechanical photothermal sensing toward a full NIR spectro- and polarization-microscopy technique. With a silicon nitride nano-optomechanical drum resonator as a sensitive thermometer, individual gold nanorods are localized and their spectra and polarization features are fully characterized, additionally shedding light on their interaction with the nanoresonator itself. Among the huge variety of nanoparticle shapes and materials, gold nanorods occupy a relevant position in gas- and liquid-phase chemical detection as well as a sensing platform for biomolecules^21,22^ or as photothermal heating sources.^23^ In the present study, the plasmonic properties of such nano-objects are analyzed and their corresponding plasmonic damping mechanisms unraveled, showing also a good agreement with finite element method (FEM) simulation results. The performance of our approach is then compared with the other state-of-the-art single molecule and particle techniques in terms of normalized SNR, showing the capabilities offered by nanomechanical photothermal spectroscopy with its superior SNR.

## Experimental Methods

The 50 nm thick, square (1 mm side-length) nano-optomechanical drumhead resonator is operated at room temperature under high vacuum conditions (*p* < 10^–5^ mbar) in order to reduce air damping and eliminate heat dissipation by convection.^7^ The 5 × 5 mm^2^ resonator chip is connected to the vacuum chamber, which acts as a thermal sink, via a metallic holder. In between, a piezoelectric element (PA2JEW, Thorlabs, Inc.) is mounted to actuate the nanomechanical resonator.

**Figure 1:**
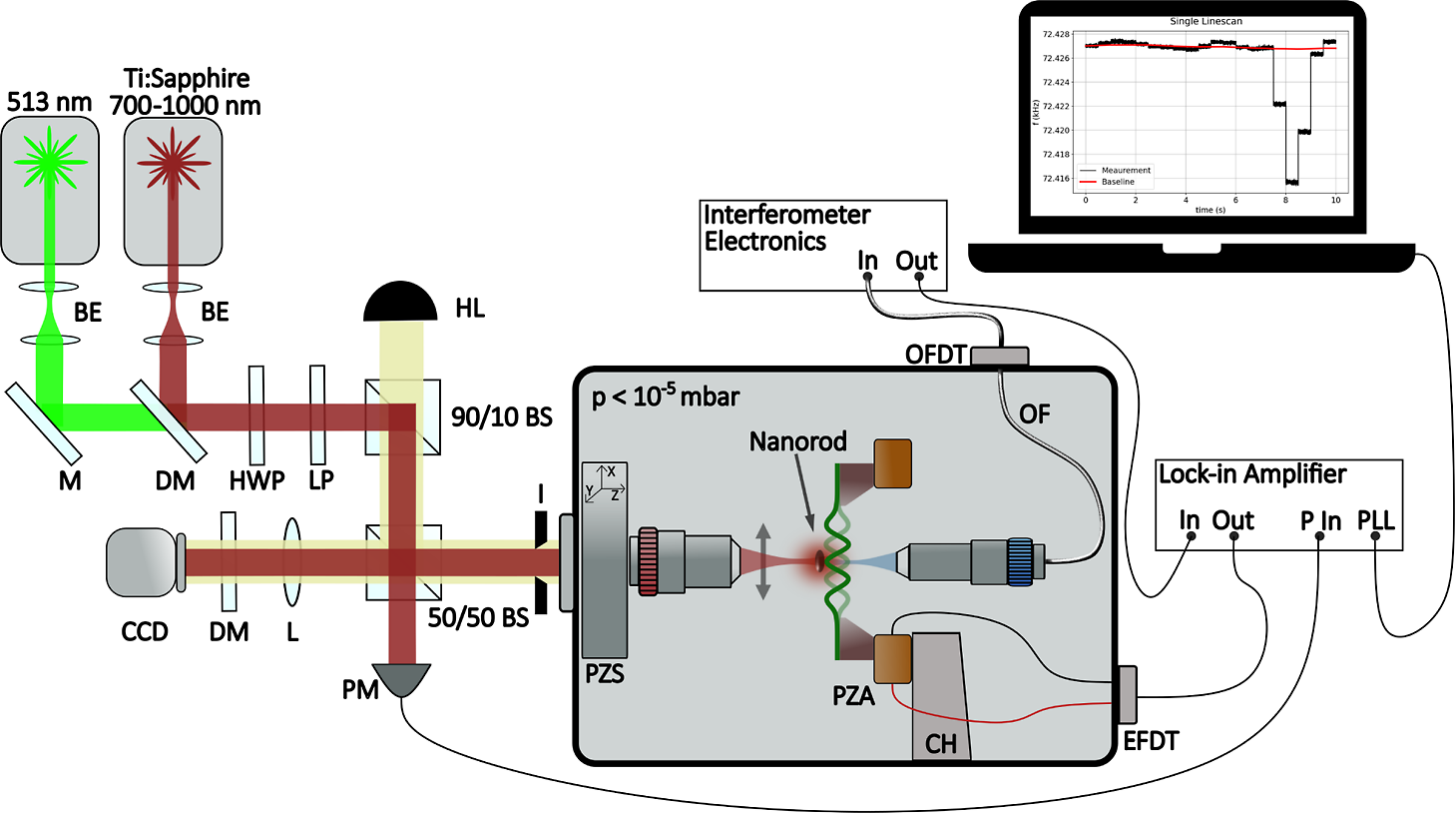
Schematics of the operating setup. The drum resonator is actuated in a vacuum (*p* < 10^–5^ mbar) by a piezoshaker. The displacement is read out by a Fabry–Perot interferometer (blue laser). The interference signal is processed and sent to the lock-in amplifier which records the frequency. The scanning lasers (red and green) are used to generate the photothermal signal by plasmonic excitation of the nanorod. BE: beam expander. BS: beam splitter. CCD: charge-coupled device camera. CH: chip holder. DM: dichroic mirror. EFDT: electrical feedthrough. I: iris. L: lens. LP: linear polarizer. HL: halogen lamp. HWP: half-waveplate. M: mirror. OF: optical fiber. OFDT: optical feedthrough. PM: power meter. PZS: piezo-stage.

The nanoresonator’s displacement is measured with a Fabry–Perot interferometer (Attocube IDS3010)^24,25^ (see Figure 1). The mechanical resonance frequency is recorded with a phase-locked loop tracking scheme (HF2LI, Zurich Instrument).

The operating setup is equipped with a green laser at 513 nm (Toptica TopMode) and a Ti:sapphire laser (M Square SolsTiS) with a tunable wavelength in the range of 700–1000 nm, used as probe beams to photothermally excite both the longitudinal (LSPR) and transverse plasmonic resonances (TSPR) of each nanorod. In fact, every time one of the two lasers is scanned across the central area of the drum and hits the sample, the corresponding light absorption results in local heating, reducing the stress of the nanoresonator and ultimately resulting in a detectable resonance frequency detuning.^7,12,15^

For the scanning laser probes, long working distance 50× objectives are used (N.A. = 0.42, M Plan Apo NIR, Mitutoyo in the NIR range; N.A. = 0.55, M Plan Apo, Mitutoyo in the VIS range). The laser’s polarization angle is controlled by means of a linear polarizer in the optical beam path. Raster scanning is made possible by a closed-loop piezoelectric nanopositioning stage (PiMars, Physikinstrumente). The probe laser power *P*_0_ is continuously recorded via a powermeter (S120C, Thorlabs, Inc.). The analyte is sampled by spin-casting a drop of diluted solution containing the nanorods onto the resonator after filtering through a 200 nm pore size PTFE membrane syringe filter (Acrodisc, Sigma-Aldrich) to prevent particle aggregation. As already shown,^7^ NEMS-based photothermal sensing enables the measurement of pure optical absorption of the sample, facilitating a comprehensive characterization of its absorption cross-section
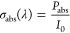
1with *I*_0_ being the peak irradiance of a Gaussian laser beam and *P*_abs_ the absorbed power by the sample. The former is a function of the input laser power *P*_0_
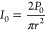
2with *r* being the beam radius, which is always characterized by the knife-edge method before each measurement.^26,27^ The latter can be calculated from the measured resonance frequency shifts, assuming full thermalization
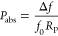
3where *f*_0_ is the resonance frequency, Δ*f* is the frequency shift, and *R*_P_ is the relative power responsivity (W^–1^). For the drumhead resonators used in this work, a responsivity of *R*_P_ ≃ 10^4^ W^–1^ has been measured (see Supporting Information Section S5). It is also worth noting that full thermalization refers to the steady-state condition: the measurement time is longer than the thermal time constant of the resonator (τ_meas_ > τ_th,res_) to guarantee complete tracking of the resonator frequency shift per absorption event. For a 1 mm side-length drumhead resonator, a τ_th,res_ ≃ 30 ms is measured with the 90/10 method.^28^

It is also worth mentioning that the thermal time constant of the nanorods analyzed here can be estimated for a continuous-wave illumination as^9^
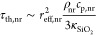
4with *r*_eff, nr_ being the effective radius of a spherical nanoparticle of volume equal to the corresponding nanorods’ volume, ρ_nr_ the mass density of gold (19,300 kg/m^3^), *c*_p,nr_ the gold’s specific heat capacity at constant pressure (129 *J*/(K kg)), and  the silica coating’s thermal conductivity (1.3 W/(K m)). For individual nanorods, thermal time constants on the order of τ_th,nr_ ≃ 40–60 ps can be estimated from eq 4 (see Supporting Information Section S6), 9 orders of magnitude smaller than the mechanical resonator, therefore not being the limiting factor for the measurement time.

## Results and Discussion

**Figure 2:**
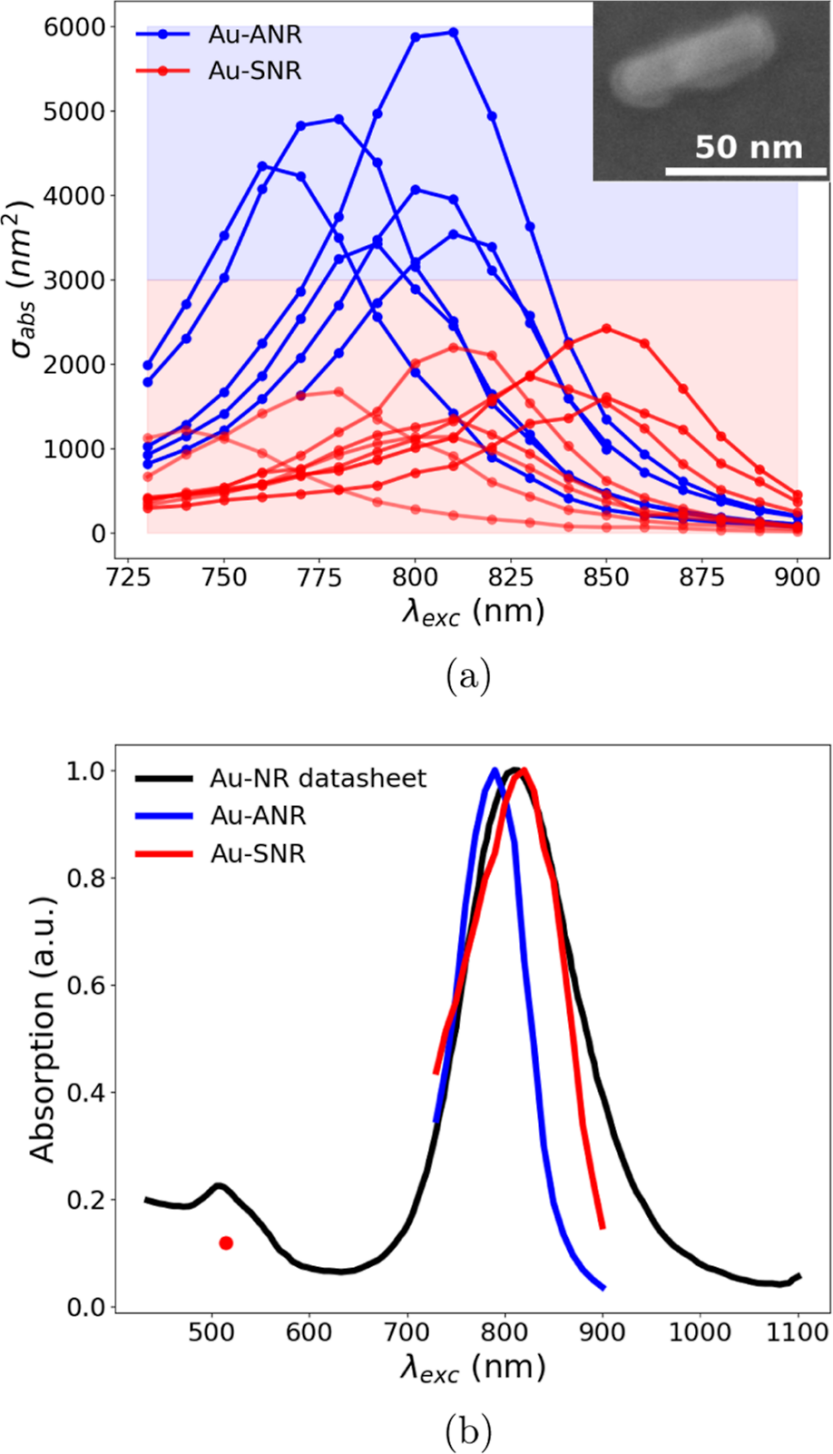
(a) Measured absorption cross-section spectra of single nanorods (Au-SNR, red curves) and small nanorod aggregations (Au-ANR, blue curves), showing the heterogeneity characterizing these samples, mainly caused by the particle size dispersion. Inset: SEM micrograph of a single silica-coated gold nanorod landing on the drum resonator. (b) Red curve and dot: renormalized sum of the measured absorption cross-section spectra of single nanorods (a); blue curve: renormalized sum of the absorption cross-sections of the nanorods aggregation; black curve: ensemble average absorption spectrum given by the datasheet.

The nanorods analyzed here have lengths *L*_nr_ in the range of ca. 38–48 nm, radial diameters *w*_nr_ in the range of 9.5–11.5 nm, and silica coating with a thickness in the range of 18–22 nm (Sigma-Aldrich silica-coated gold nanorods) (Figure 2a, inset: the majority of the nanorods have been SEM imaged by deposition of 10 nm thick gold layer on top of them to reduce any possible charging effect). Their optical properties in the visible and near-infrared range are characterized by surface plasmon resonances (SPR), that is the electromagnetic coupling between an impinging light and the collective motion of the conduction band electrons.^8,29^ The main interest in gold nanorods lies in their large SPR amplitudes and broad spectral tunability.^30–42^ In the specific case where a coating is present, depending on its thickness, SPR features will be affected more or less by the environment. Indeed, the plasmonic response is sensitive to its surroundings on the spatial range of the order of the nanorod diameter, the region where the field enhancement takes place^34,43–45^ (Supporting Information Figure S1). In the present study, a silica coating of roughly 20 nm is thin enough for the SPR to remain sensitive to both the coating and the surrounding medium, but within a reduced magnitude.^44^

Figure 2a shows the measured absorption spectra of different, individual silica-coated gold nanorods (Au-SNR) indicated by the red curves, together with some aggregations of a few units (Au-ANR) indicated by the blue curves. Differentiating these two photothermal responses is aided by FEM electromagnetic simulations (for an overview of how aggregations can be differentiated from individual nanorods, see Supporting Information Section S2). For each spectrum, the polarization of the probe beam (here Ti:sapphire laser) was set to maximize the absorption in the wavelength range of 700–900 nm. In fact, the nanorods analyzed here present maximum absorption in the range of ca. 790–830 nm (see Figure 2b, black solid curve) due to LSPR excitation whenever the laser polarization is parallel to their long axis. With the nanomechanical photothermal technique, nanoparticle heterogeneity can be investigated, revealing more than the information obtained in ensemble average measurements. Here, the heterogeneity is mainly due to the size dispersion of the particles, as stated by the vendor,^46^ and it relates both to the LSPR spectral position λ_LSPR_ and the absorption cross-section amplitude σ_abs_(λ_LSPR_). Moreover, the latter has a strong dependence on the substrate, specifically on its thickness and optical properties (see Figure 5; further details in Supporting Information Section S3).

The red solid line in Figure 2b indicates the corresponding renormalized sum of the responses from individual nanorods (Au-SNR in Figure 2a), showing a very good match with the reference spectrum given in the datasheet (black solid line), recovering a typical ensemble average absorption spectrum.^30,32^ The ensemble Au-SNR wavelength λ_LSPR_ is measured to be 809 nm, which is close to the nominal value of 808 nm.^46^Figure 2b also shows the renormalized spectrum considering only responses from nanorod aggregations (Au-ANR, blue solid line). For this spectrum, an Au-ANR wavelength of 786 nm is extracted, corresponding to a blue-shift of 2.8% from the Au-SNR wavelength. As shown by Jain et al.,^47^ such a blue-shift occurs in nanorod aggregations of two or more units assembled in a side-by-side orientation, for a polarization parallel to their long axis. As the authors reported, the strength of this shift depends on the interdistance between the nanorods involved, on their aspect ratios, the relative orientational angle, and the number of units considered. For the spectral distributions seen in Figure 2 (blue curves), we expect these signals to originate from side-by-side assembled nanorod aggregations.

The individual nanorods have also been measured with a wavelength of 513 nm to excite the TSPR (red dot in Figure 2b). As expected, the absorption at this wavelength is roughly 1 order of magnitude smaller than LSPR, due to the overlap between the transverse localized plasmonic resonance and the electronic interband transitions, which start to arise at 2.4 eV ca. in gold,^48–50^ ultimately resulting in enhanced plasmonic damping.

**Figure 3:**
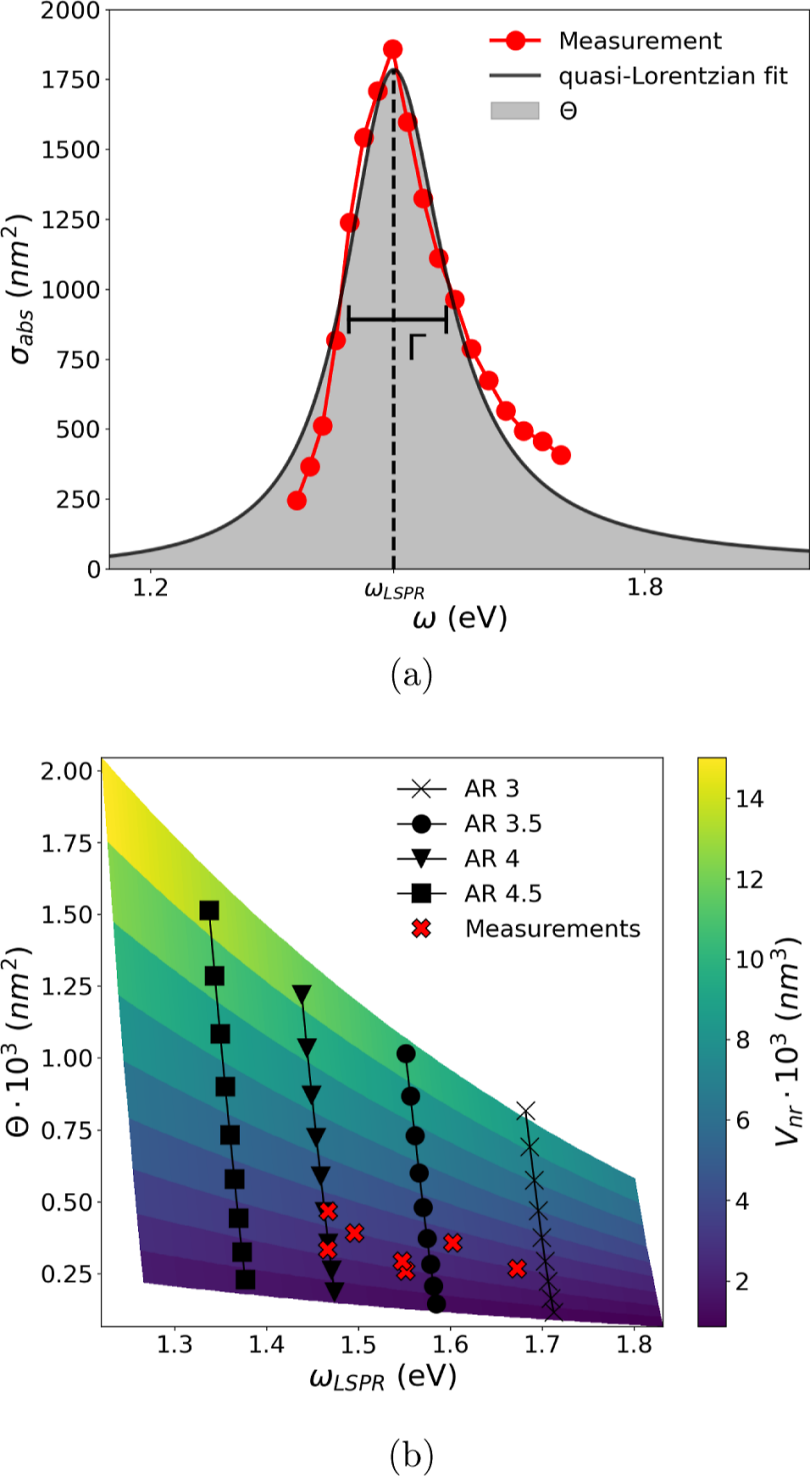
(a) Example of fitting of the measured absorption cross-section with a quasi-Lorentzian function (eq 5). (b) Calibration surface plot of the nanorod volume, constructed according to the procedure developed in refs (41) and (42).

All the measured LSPR spectra of the individual gold nanorods have then been fitted with a quasi-Lorentzian function
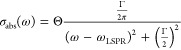
5with ω_LSPR_ = ℏ*c*/(λ_LSPR_) being the longitudinal surface plasmon resonance energy (eV), Γ the overall plasmonic resonance line width (eV), and Θ the integrated oscillator strength (nm^2^).^41,42^Figure 3a shows an example of the fitting of the measured absorption cross-section (black curve), together with the quantities entering eq 5. To gain better insight into the spectral properties of the measured individual nanorods and how these are affected by the sample size dispersion, the volume *V*_nr_ and aspect ratio AR = *L*_nr_/*w*_nr_ for each individual gold nanorod have been extracted, following the same procedure developed in refs (41) and (42) ultimately enabling the quantification of the different plasmonic damping contributions to the line width Γ. The procedure exploits the dependence of ω_LSPR_ and Θ on the nanorod’s volume and aspect ratio, and it works as follows.

First, a series of spectra is calculated with the help of FEM and the T-matrix method for different nanorod radial diameters *w*_nr_ (8–16 nm) and aspect ratios AR (2.5–5), and fitted with eq 5 (Supporting Information, Sections S1 and S2). Second, a calibration surface plot of the nanorod volume, such as the one shown in Figure 3b, is constructed from the simulation results, where the resonance energy ω_LSPR_ and the integrated oscillator strength Θ are treated as independent variables. The red crosses in Figure 3b represent the measurements of individual nanorods. From the calibration plot, the volume and aspect ratio of each absorber are extracted.^41,42^ It is worth noting that the resonance energy ω_LSPR_ strongly varies with the aspect ratio (AR), red-shifting for higher AR, yet presenting a small dependence on the nanorod radial diameter *w*_nr_, and red-shifting for increasing *w*_nr_, as expected.

**Figure 4:**
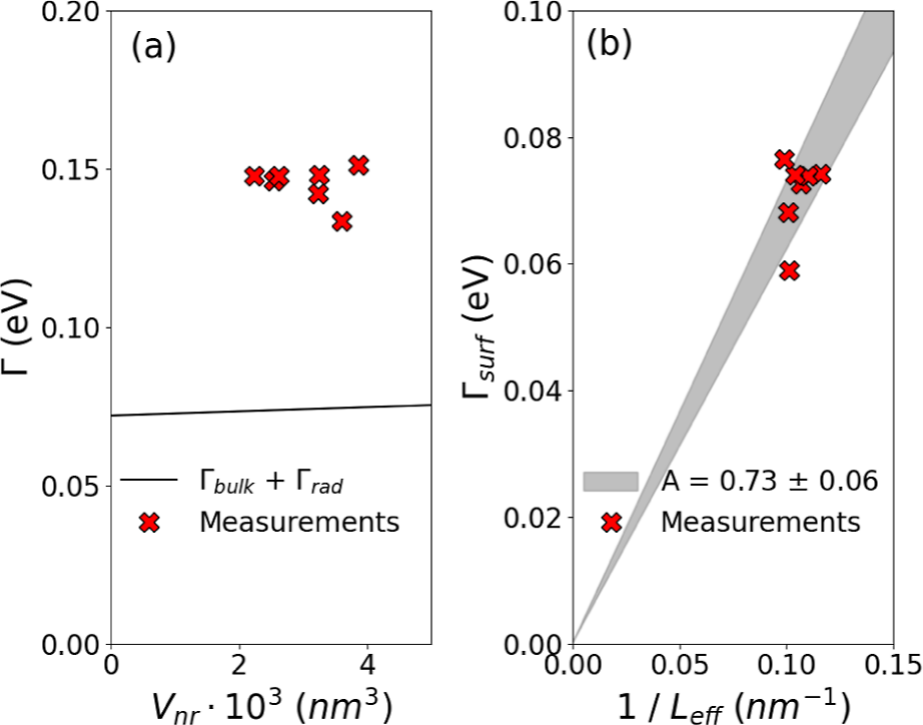
(a) Measured LSPR line width Γ (red crosses) as a function of the nanorod volume *V*_nr_. For comparison, the contribution of Γ_bulk_ + Γ_rad_ is plotted (black curve). (b) Extracted electron surface scattering Γ_surf_ = Γ – (Γ_bulk_ + Γ_surf_) for the measured individual nanorods (red crosses) as s function of the inverse effective length 1/*L*_eff_, compared with the theoretical model Γ_surf_ = *v*_F_*A*/*L*_eff_. A value of *A* = 0.73 ± 0.06 is found for these nanorods.

Various optical scattering phenomena of conduction band electrons can contribute to the broadening of LSPR resonance in metallic nanorods (see also Supporting Information Section S1).^41,42^ Briefly, electron bulk scattering (Γ_bulk_), due to electron–electron, electron–phonon, and electron–defect interactions, is always present and constant across the wavelength considered here (in gold structures, Γ_bulk_ = 73 meV). Part of the absorbed electromagnetic energy can also be re-emitted to the environment, constituting a radiative dissipation path (Γ_rad_). This mechanism is directly proportional to the particle volume Γ_rad_ = η/π *V*_nr_, with a defined proportionality constant η/π = 6.6 × 10^–7^ eV nm^–3^ (see Supporting Information Figure S2b and Section S2).^40,50^Figure 4a displays the measured LSPR line widths Γ (red crosses, 130–150 meV), together with the two contributions Γ_bulk_ + Γ_rad_ for comparison (black curve). Because of the large mismatch, it is evident that the observed broadening cannot be explained by radiative damping only, as expected. Indeed, the reduced volume of the nanorods analyzed here makes this contribution small, in contrast to what has been observed in spherical gold nanoparticles.^50^

To explain the experimental line widths (Γ) obtained, electron surface scattering (Γ_surf_) has to be taken into account.^40–42^ Its contribution is evaluated via subtraction Γ_surf_ = Γ – (Γ_bulk_ + Γ_surf_), and the results are displayed in Figure 4b (red crosses).

Γ_surf_ is mainly due to quantum confinement effects. The smaller the nanostructure, the more pronounced its contribution to broadening, as demonstrated by these measurements. In a first approximation, it can be defined as Γ_surf_ = *v*_F_*A*/*L*_eff_, *v*_F_ = 1.4 × 10^6^ m/s being the Fermi velocity; *A*, an experimentally determined proportionality constant; and *L*_eff_ = 4*V*_nr_/*S*_nr_, an effective electron path length (see Supporting Information Sections S1 and S2). From the experiments, a value of *A* = 0.73 ± 0.06 has been determined (Figure 4b), close to what has been reported in literature.^40,41^ This corroborates the evidence that electron surface scattering is a major source of damping in this context.

**Figure 5:**
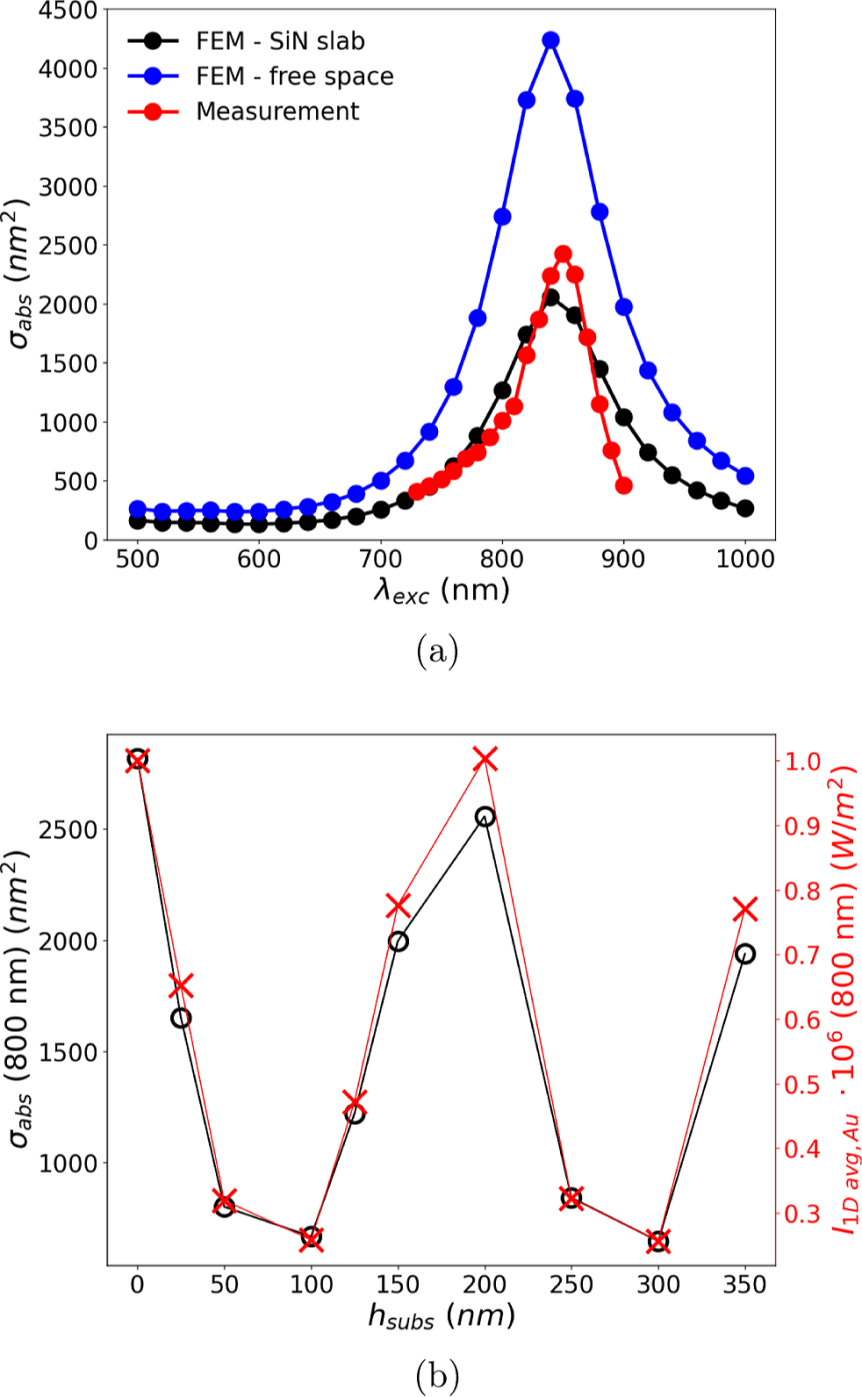
(a) Measured absorption cross-section of an individual nanorod (red dots), compared to FEM simulated absorption spectrum in the presence of the silicon nitride substrate (black dot) and in free space (blue dots), obtained for nanorod dimensions of L = 48 nm, *r* = 6 nm, with the silica coating thickness of 20 nm. (b) FEM absorption cross-section at 800 nm wavelength for the same nanorod (black empty dot and solid line) and 1D averaged FEM electromagnetic intensity in the vicinity of the gold core in the presence of the substrate only (red crosses and solid line), for different silicon nitride slab thicknesses.

Figure 5a focuses on the nanomechanical photothermal spectrum of another individual nanorod (red dots). This specific sample has maximum absorption at λ ≈ 840 nm with a cross-section of σ_abs_ ≈ 2.5 × 10^–15^ m^2^, close to what is reported in the literature.^33–35^ The measurements are compared with FEM simulations (black and blue dots, Figure 5a), showing good agreement with data (black dots). Indeed, FEM approaches offer the possibility to evaluate the absorption and scattering cross-sections of an arbitrarily shaped particle.^51–57^

Figure 5a also clearly shows how the presence of the silicon nitride resonator ultimately affects the absorption cross-section of the nanorod under study. The FEM analysis in the presence of the substrate (black dots) closely matches the measured absorption spectrum, whereas the FEM analysis conducted in free space in the absence of the slab does not (blue dots). More precisely, at the LSPR wavelength (840 nm), the absorption cross-section results are roughly half of the free space case (≈2 × 10^–15^ m^2^ in the presence of the slab and ≈4.2 × 10^–15^ m^2^ in air). In contrast, the LSPR wavelength and line width Γ are weakly affected by the presence of the substrate (≤1% difference for both quantities). In general, a dielectric substrate underneath a metal nanoparticle screens the electromagnetic restoring force acting on plasmon oscillations. This screening can be modeled qualitatively as a nanoparticle image with a reduced number of charges, whose electromagnetic strength is determined by the nanoparticle–substrate interdistance and the slab dielectric permittivity.^56^ The interdistance of 20 nm (silica coating thickness) and the relatively small refractive index of low-stress silicon nitride (whose spectral distribution has been taken from ref (58)) give a reason for this weak effect.^44,56^ To better understand the role played by the silicon nitride slab, FEM simulations were performed at a single wavelength (800 nm) for different thicknesses *h*_subs_.

In Figure 5b, the absorption cross-section follows a periodic pattern for an increasing substrate thickness. This modulation perfectly follows the variation in intensity at the interface of air-silicon nitride in the vicinity of the nanorod. In fact, the electromagnetic losses *Q*_h_ due to absorption are directly proportional to the intensity of the electric field, *Q*_h_ ∝ |**E**(**r**)|^2^ (Supporting Information eq S1). This intensity modulation is due to the interference occurring between the input electric field and the reflected light from the slab, whose magnitude depends on the thickness and refractive index. Here, the calculated intensity value is an average over its spatial distribution in the proximity of the nanorod, however, in the absence of the nanorod and with only the presence of the substrate (Supporting Information Figure S4). For the thickness used in this study (50 nm) along with the considered wavelengths (730–900 nm) and the refractive index of silicon nitride,^58^ no interference inside the slab is present as is the case in ref (59). There, 1 μm thick silicon cantilevers served as an optical cavity for specific wavelengths in the VIS range, modulating the scattering of deposited 100 nm gold nanoparticles. In this study, however, the most relevant interference occurs at the interface between the free space and the substrate. Therefore, by controlling the substrate refractive index and thickness, it is possible to tailor the absorption spectrum of individual nanoabsorbers.

**Figure 6:**
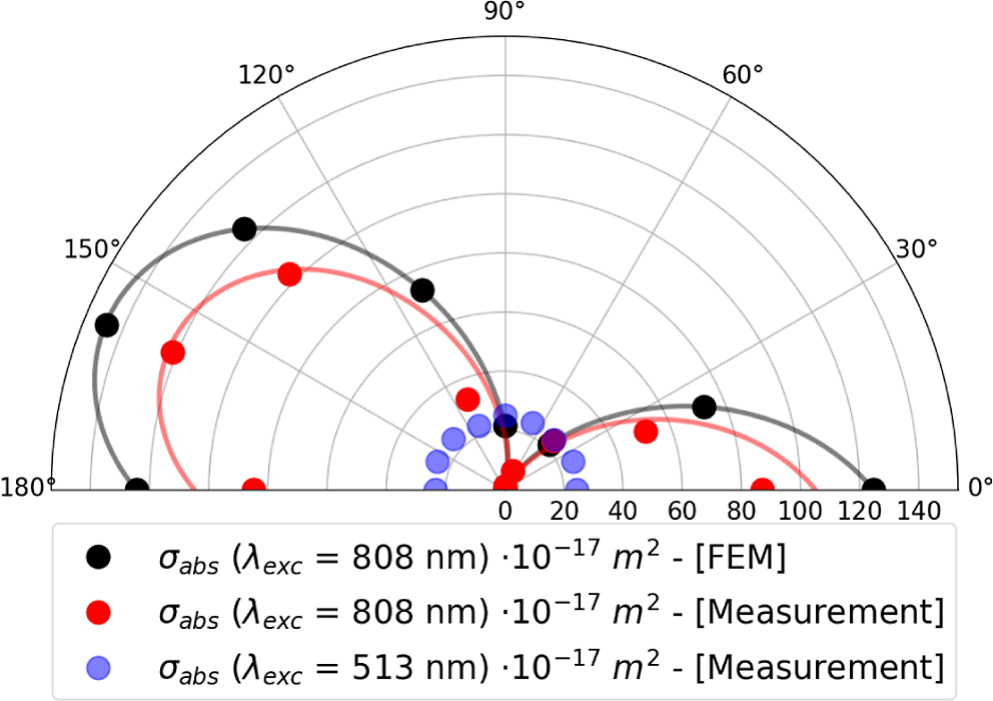
Polar plot of the absorption cross-section of an individual nanorod measured at λ_exc_ = 808 nm as a function of polarization angle θ_pol_ (red dots). The ratio between the absorption cross-section for a polarization parallel to the nanorod long-axis (θ_pol_ ≈ 157.5°) and perpendicular to it (θ_pol_ ≈ 90°) is roughly σ_abs,∥_(808 nm)/σ_abs,⊥_(808 nm) ≈100. FEM simulations show good agreement with the measurement (black dots). Both the red and black solid curves represent the cos^2^(θ) pattern. Blue dots represent nanomechanical photothermal measurements at λ_exc_ = 513 nm.

**Figure 7:**
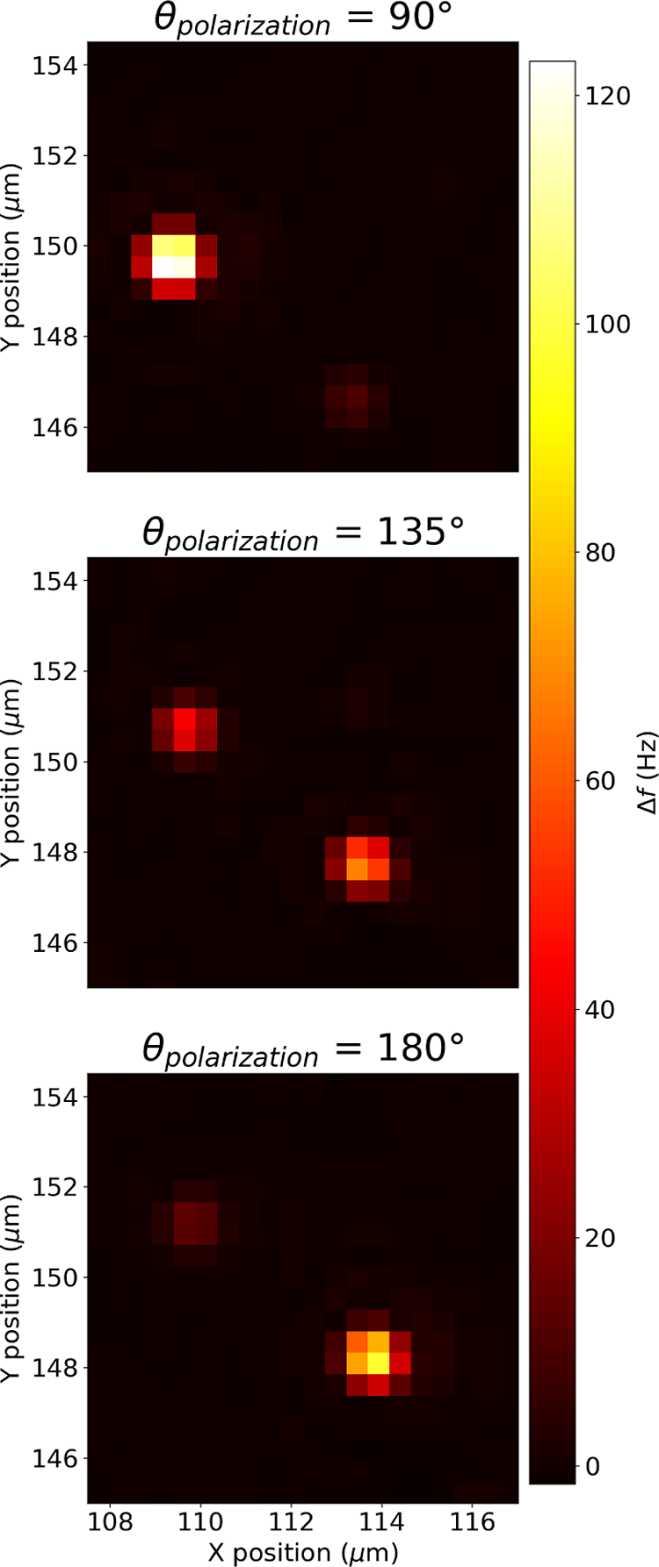
2D maps of the same region at λ_exc_ = 808 nm for three different polarization angles θ_pol_: 90, 135, 180°. The two responses are from two individual nanorods. For the perpendicular polarizations, 90 and 180°, the absorbers behave in an opposite way, meaning that they are almost perpendicular to each other, while absorbing almost the same amount of light for the central map (θ_pol_ = 135°).

For these nonspherical nanoparticles, absorption is strongly polarization dependent, as clearly seen in Figure 6 and 7. Figure 6 shows how the absorption cross-section varies with the laser polarization angle for an individual nanorod, with the red dots representing the nanomechanical photothermal measurements close to its LSPR (λ_exc_ = 808 nm). Each point is acquired by changing the polarization of the scanning laser probe with steps of 22.5° by means of a half-waveplate (HWP) and a linear polarizer while maintaining the same laser input power. The ratio between the absorption cross-section for a polarization parallel to the nanorod long-axis (θ_pol_ ≈ 157.5°) and perpendicular to it (θ_pol_ ≈ 90°) is roughly
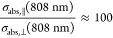
6

This high polarization contrast gives us therefore an insight into the absorption efficiency achievable in this nanoabsorber upon control of the incident laser polarization. The absorption efficiency for the parallel case is *Q*_abs,∥_ ≈ 3.64, while for the perpendicular case *Q*_abs,⊥_ ≈ 0.03. Here, the nanorod has an area *A*_nr_ ≈ 3.29 × 10^–16^ m^2^, calculated using the sizes extracted from FEM simulations. The measurements have been compared also to the FEM simulations (black dots), showing a good match. It is worth noting how the measurements follow the typical pattern σ_abs_(λ, θ_pol_) = σ_abs,∥_(λ)cos^2^(θ_pol_) expected for perfect dipoles.^30,37,60^

For comparison, Figure 6 also shows the nanomechanical photothermal signal at λ_exc_ = 513 nm (the blue dots). The TSPR shows almost no polarization contrast since it starts to overlap with polarization-independent electronic transitions in gold.^48^ For this reason, the plasmonic damping increases, causing the transverse plasmonic resonance strength to be less than the longitudinal strength.

Nanomechanical photothermal spectromicroscopy also allows the precise determination of the orientation on the substrate of different nanoabsorbers, as seen in Figure 7. 2D maps of the same region on the drum resonator for three different polarization angles θ_pol_ (90, 135, 180°) are acquired at λ_exc_ = 808 nm. The two responses are from two individual nanorods, the absorption amplitude of which varies as a function of the laser polarization. Focusing the attention on the two perpendicular polarizations, 90 and 180°, the two absorbers behave in an opposite way, meaning that they are almost perpendicular to one another, while absorbing almost the same amount of light for the central scenario (θ_pol_ = 135°). Nanomechanical photothermal spectromicroscopy can be therefore employed in the analysis of the more complex optical features, for nanorods and more exotic structures, like plasmon-assisted optical chirality in metallic nanoparticles.^38,61,62^

**Figure 8:**
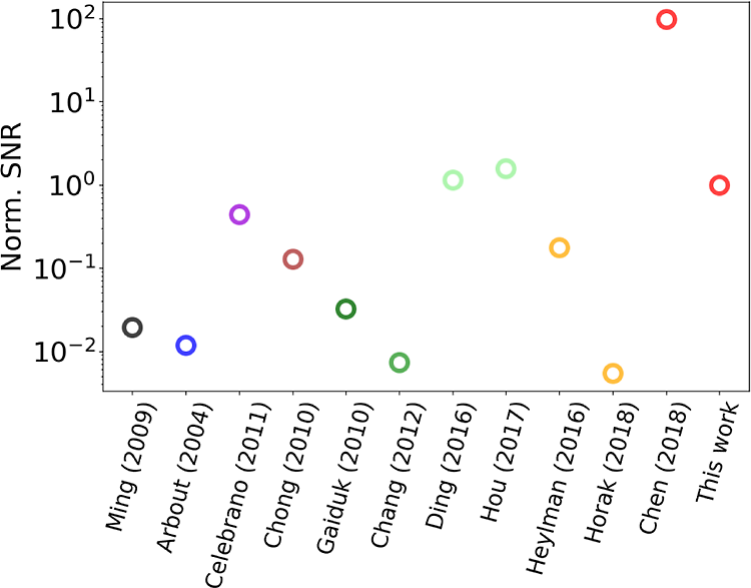
SNR comparison between different single-molecule absorption sensing techniques. Black: UV–vis extinction;^60^ blue: spatial modulation spectroscopy;^64^ violet: extinction microscopy plus balance detection;^3^ brown: ground-state depletion microscopy;^5^ dark green: photothermal contrast microscopy (PCM) with glycerol;^16^ green: PCM with thermotropic liquid crystal 5CB;^17^ light green: PCM with near-critical Xe;^18,19^ orange: optical microresonator;^37,65^ red: nanomechanical photothermal microscopy.^7^

Finally, to further stress the advantages offered by nanomechanical photothermal spectromicroscopy compared to other label-free single-particle and molecule spectromicroscopy techniques, a comparison between SNR of different approaches is carried out in the following way^63^
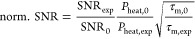
7for the sake of completeness, eq 5 takes into account, together with the SNR itself, also the power absorbed by the sample under study *P*_heat_ and the time constant of the experiment τ_m_. The reference values of the three quantities used for normalization (labeled with the subscript 0) correspond to the value obtained for the individual nanorod of Figures 5 and 6. The calculations are plotted in Figure 8, for which the experimental values extracted from the listed references are used (for the used values, see Supporting Information Section S4).^3,7,17–19,32,37,60,64,65^

The different colors correspond to different techniques (see caption). It is worth noting that the nanomechanical photothermal approach of ref (7) (first red empty dot) presents the highest SNR, followed by photothermal contrast microscopy with near-critical Xe^18,19^ and this work. More precisely, the difference of 2 orders of magnitude between this work and ref (7) lies in the different prestress of the nano-optomechanical resonator. There, oxygen plasma treatment is exploited to reduce the stress of the resonator, with the aim to improve its relative power responsivity *R*_P_ to detect single Atto 633 molecules. Here, there has been no need to further reduce the stress due to the already high sensitivity of the resonator used for the nanorods detection. For the sake of completeness, it is worth mentioning that another interesting approach for improvement of the power sensitivity consists in the patterning of the drumhead resonator, for example with a trampoline design, reducing the heat dissipation via heat conduction through the anchoring points.^66–68^ Still, this work shows the superior capabilities of nanomechanical photothermal spectromicroscopy over a wide range of label-free absorption techniques.

## Conclusions

In conclusion, the optical absorption cross-section of individual silica-coated gold nanorods in the NIR range has been measured and quantitatively characterized using nanomechanical photothermal spectroscopy and microscopy, likewise elucidating their polarization features. With this approach, where the substrate acts as a temperature sensor, it is possible to shed light on the variations in nanoabsorbers’ properties to investigate concealed heterogeneity, as expected for these complex systems, as well as their reciprocal intercoupling, which opens up a wealth field of research by its own. It has also been shown that these nanorods present, on one hand, a pronounced plasmonic electron-surface scattering, broadening their LSPR in conjunction with bulk scattering. On the other hand, a strong polarization contrast on the order of a few hundred has been observed. The interaction between the silicon nitride slab and the nanorod has been also investigated, consisting of a modulation of its absorption strength over the whole considered spectrum while weakly affecting the plasmonic resonant energy and its broadening. This result underlines the importance of taking into account the interaction of the substrate in all of the experiments where a support is used for spectroscopic measurements.

Primarily, this work demonstrates the capabilities of nanomechanical photothermal NIR spectromicroscopy for localizing individual nanoparticles, obtaining their plasmon spectra, and resolving their polarization features, pushing our understanding of the light-matter interaction at the nanoscale level. A comparison among the different label-free single-molecule techniques shows that nanomechanical photothermal sensing presents a superior SNR within a less complex experimental setup and measurement procedure.

## Supporting Information Available

FEM simulations details (computational approach, gold dielectric function model); LSPR line width (measurements and modeling); FEM substrate analysis; table with all the parameters used for the SNR comparison; relative power responsivity characterization; nanorod thermal time constant estimation; SEM sample size distribution (PDF)
