# Supplementary material for: Nanomechanical Photothermal Near Infrared Spectromicroscopy of Individual Nanorods
Source: ACS Photonics. 2023 Sep 20;10(10):3730–9. doi: 10.1021/acsphotonics.3c00937 (PMC10588552; doi:10.1021/acsphotonics.3c00937)
Supplement: Supplementary file 1 — ph3c00937_si_001.pdf [file ph3c00937_si_001.pdf]

Supporting Information: Nanomechanical Photothermal Near Infrared Spectromicroscopy of Individual Nanorods

Kostas Kanellopulos, Robert G. West, and Silvan Schmid*

Institute of Sensor and Actuator Systems, TU Wien, Gusshausstrasse 27-29, 1040 Vienna, Austria.

E-mail: silvan.schmid@tuwien.ac.at

Contents

Supporting Section S1. FEM nanorod simulations.

Supporting Section S2. LSPR linewidth and difference between individual nanorods and aggregation.

Supporting Section S3. Silicon nitride substrate.

Supporting Section S4. Signal-to-Noise ratio comparison.

Supporting Section S5. Relative power responsivity characterization.

Supporting Section S6. Nanorod thermal time constant estimation.

Supporting Section S7. SEM sample size distribution.

Supporting Section S1. FEM nanorod simulations

The finite element model of the silica-coated gold nanorods lying on a silicon nitride substrate was built using the commercially available FEM software COMSOL Multiphysics (COMSOL Inc., Burlington, MA), version 5.5. The shape of the nanorods was extracted from SEM measurements, and each single nanorod was mimicked by a cylinder of length L_{nr}, radius r_{nr}, terminated with hemispherical caps with the same radius (see Figure S1).[1] In this way, concerning the nanorod gold core, the only parameters to be changed for comparison between measurements and simulations are the length L_{nr} and the radius r_{nr}. It is worth mentioning that in prolate ellipsoids, the aspect ratio plays a central role in determining the wavelength λ_{SPR} and amplitude $\sigma_{abs}(\lambda_{SPR})$ of the longitudinal SPR.[2]

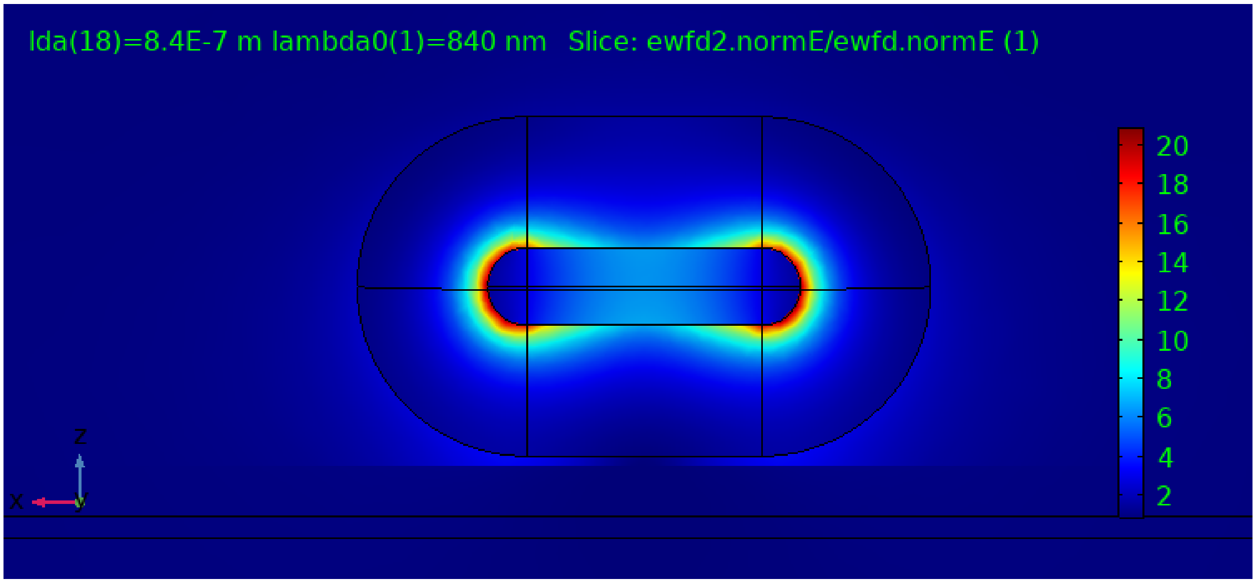

Figure S1: Simulated field enhancement assuming a laser beam with $\lambda = 840$ nm polarized parallel to the long axis of the nanorod. It takes place in the region surroundings the hemispherical caps of the nanorod, of the order the particle diameter.[3,4]

The FEM simulation resolves the spatial distribution of the electromagnetic field on the defined physical domain, $\mathbf{E}(\mathbf{r})$, using both the full and scattered field formulation in two successive steps.[6,7] In the first step, the interaction between an incident electromagnetic plane wave and the substrate is computed with the full field formulation in the absence of

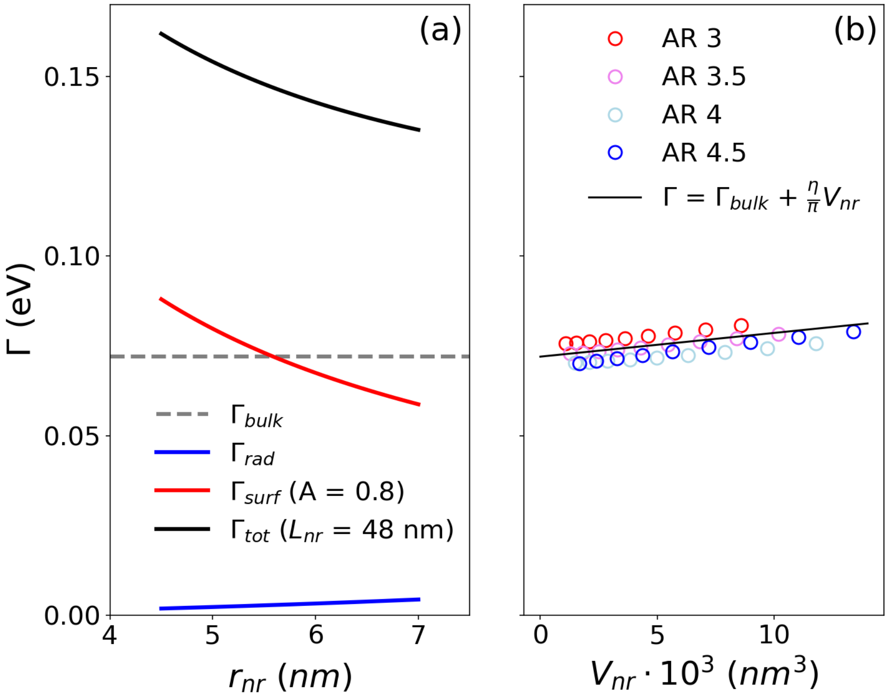

Figure S2: (a) Analytical model of the damping mechanisms behind the broadening of the nanorod LSPR resonance. Γ_{bulk} represents the mean bulk-like electron scattering rate (grey dashed line); Γ_{surf} represents the electron-surface scattering rate (red solid line); Γ_{rad} represents the radiative damping (blue solid line). The black solid line indicates the overall damping Γ_{tot}, sum of the three contributions. Electron-bulk and surface scattering are the major source of plasmonic damping, with the radiative one being almost two orders of magnitude smaller. (b) Simulated LSPR linewidths Γ for different aspect ratios AR as a function of the nanorods volume V_{nr}. For these results, only the bulk dielectric function for gold (Reference 5) was used. The black curve indicates a linear fitting, whose gradient value is $\eta/\pi = 6.6 \cdot 10^{-7}\ eV \cdot nm^{-3}$.

the nanorod. The incident electric field is set to be parallel to the nanorod's long axis. The results of this simulation are then used as input background electric field for the second step, where the scattered field formulation is exploited to calculate the overall electric field inside the nanorod and its corresponding absorption cross-section. Perfectly matched layers (PML) are used to avoid artificial reflections at the simulation domain boundaries and act as an anisotropic absorbing layer. Both PML and mesh setting parameters are optimized to show very good agreement between spherical gold nanoparticles and Mie theory predictions in free space, both for the absorption and scattering cross-sections. In Figure S1 the electric field enhancement in the nanorod and its surroundings is plotted, showing how the electric field is strongly concentrated at the nanorod's tips for the first order LSPR, giving rise to the so-called hotspots. Once the electromagnetic field is computed, the nanorod's absorption cross-section is obtained by integrating the absorbed power density due to resistive losses Q_h over the nanorod's volume V_{nr} (including both the gold core itself and its silica coating), and dividing it by the incident planewave light intensity I_0:[8]

$$\sigma_{abs}(\omega) = \frac{1}{I_0} \iiint_{V_{NP}} \frac{1}{2} \omega \epsilon_0 Im(\epsilon_{NR}(\omega, \mathbf{r})) |\mathbf{E}(\mathbf{r})|^2 \, d\mathbf{r} \tag{S1}$$

ϵ_0 is the vacuum dielectric constant and $Im(\cdot)$ the imaginary part operator applied here on the dielectric function. The dielectric response of the gold core is defined starting from the bulk values for gold, ϵ_{bulk} (taken from Ref. 5), and corrected taking into account the intrinsic size effects. Here, both electron-surface scattering and radiative damping were introduced on the overall complex dielectric function for the metal core ϵ_{NR}:

$$\epsilon_{NR}(\omega, L_{eff}) = \epsilon_{bulk} + \frac{\omega_p^2}{\omega^2 + i\omega\gamma_0} - \frac{\omega_p^2}{\omega^2 + i\omega(\gamma_0 + \frac{Av_F}{L_{eff}} + \frac{\eta V}{\pi})}. \tag{S2}$$

Here, γ_0 represents the bulk-like electron scattering rate for gold; v_F indicates the electron Fermi velocity; A is a dimensionless parameter related to the details of the electron surface scattering; L_{eff} the electron mean free path confined at the surface, dependent on the sizes

of the nanorod.[3,9] Finally, V is the nanorod volume and η represents an effective radiation damping rate.[9] Therefore, the resulting broadening of the LSPR can be modelled as:

$$\Gamma_{tot} = \Gamma_{bulk} + \Gamma_{surf} + \Gamma_{rad} \tag{S3}$$

with $\Gamma_{bulk} = \gamma_0$, $\Gamma_{surf} = Av_F/L_{eff}$ and $\Gamma_{rad} = \eta V/\pi$. The parameter A is another degree of freedom used for the comparison between measurements and simulations. For the specific sizes investigated here, the radiative damping is almost two orders of magnitude smaller than the surface and bulk contributions (see Figure S2). This is due to the reduced volume compared to gold nanoparticles of the same sizes, for which it starts to be the main damping mechanism for radii > 30 nm. It is also worth noting, on one hand, that for a fixed value of A and nanorod's length, the overall damping shows a weak dependence on the nanorod's radius in the available range of values. On the other hand, for a fixed set of sizes, the plasmonic linewidth shows a strong dependence on the surface scattering parameter, A (see next section).

Simulations have been performed also to ascertain the proportionality constant η/π introduced for the radiative damping contribution Γ_{rad}. Figure S2b shows the results used for the construction of the calibration surface plot of the nanorod volumes (Figure 3b in the main text), plotted against the corresponding volumes V_{nr}. Here, only the bulk dielectric function for gold (Reference 5) has been used. It is visible that the Γ increases linearly as a function of V_{nr} with a proportionality constant $\eta/\pi = 6.6 \cdot 10^{-7}\ eV \cdot nm^{-3}$, exactly as reported in literature.[10–12]

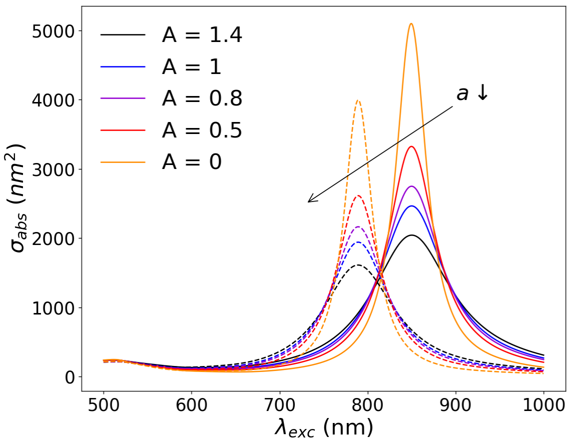

(a)

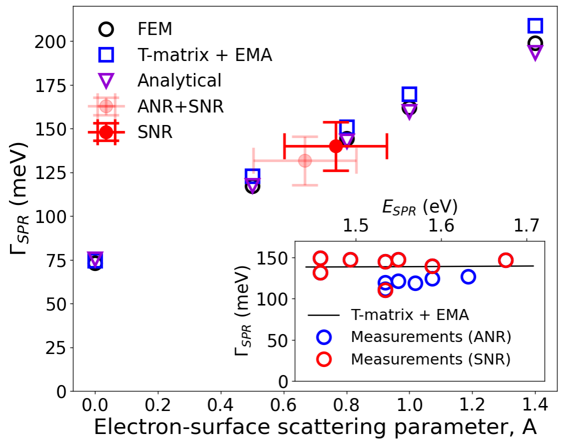

(b)

Figure S3: (a) Absorption spectra of the LSPR resonance calculated with the T-matrix method together with effective medium approximation (EMA), for different value of the parameter A, for two different aspect ratios $AR = a$ (3.5, dashed lines; 4, solid lines). (b) LSPR linewidth's dependence on the parameter A for: the analytical model (Equation (S3)), empty dark violet downward triangles; FEM, empty black circles; T-matrix plus EMA, empty blue squares; mean value and standard deviation of the measured linewidths considering single nanorods, red dot, and single nanorods plus aggregations, light red dot. Inset: linewidth as a function of the LSPR energy for the T-matrix calculation (black solid line) and measurements of single nanorods (empty red dots), and aggregations (empty blue dots).

Supporting Section S2. LSPR linewidth and difference between individual nanorods and aggregation

The nanorods LSPR resonances are measured and their linewidths quantified. These values are then compared with the analytical model introduced above in Equation (S3), together with the FEM simulations, and the T-matrix method[13] applied with the effective medium approximation (EMA),[14] for the same nanorod's shape as used in the FEM analysis. The last approach used here is not thought to be fully explanatory for the nanorod's LSPR resonances analyzed in the main text (for which FEM simulations were carried out), but it helps to understand in a simple way how the absorption spectrum changes as function of the electron-surface scattering and to differentiate between single gold nanorods and aggregations of two or more nanoabsorbers. As for the FEM, the T-matrix method requires as input the material parameters of gold (modelled exactly as for the FEM with Equation (S2)). The silica coating is taken into account by considering an effective dielectric function of the medium surrounding the nanorod, as done by Lioi et al.[14] Figure S3a shows how strongly the LSPR resonance depends on the surface scattering parameter A, for two different aspect ratios $AR = L_{nr}/(2\ r_{nr})$ (3.5 for the dashed lines, 4 for the solid lines). It is worth noting that already going from a value of 0 (bulk plus radiative damping) to a value of 0.5, a strong reduction of the absorption peak $\sigma_{abs}(\lambda_{SPR})$ is observable in both cases (35% reduction). The higher the value of A, the lower the absorption peak. It is also worth noticing that by reduction of the nanorod's aspect ratio a, the LSPR energy is blue-shifted,[15] increasing the overlap between the plasmonic-assisted resonance and the interband transitions, ultimately resulting in a reduction of the overall absorption cross-section for the same values of electron-surface scattering. The plasmonic linewidths measured in this study lie in a range comprised between ca. 110 and 150 meV, meaning that electron-surface scattering must be accounted for. By application of the analytical model on the measured plasmonic resonances, a value of A is extracted for each measurement, covering a range between 0.4 and 0.9, as seen in

Figure S3b. The spread could be due to non-uniform distribution of the silica coating, as well as surface defects of the gold core itself. Interestingly the linewidth follows linearly the change of the parameter A (blue empty triangles), allowing a simple a evaluation for the experimental values. The same behavior is observed with the FEM simulations (empty black dots) and with the T-matrix method (empty blue squares). It is also shown once again the better matching of the FEM with the analytical model compared to the T-matrix, demonstrating once again the superiority of the FEM approach. The light red dot indicates the average value of the measured linewidths (Figure S3b, inset), considering both single and aggregated nanorods, together with the standard deviations computed both for the linewidth itself and the parameter A, showing the spread of the obtained values. With an average value of 0.65 for A, one can conclude that all the spectrally resolved nanorods are strongly affected both by electron-bulk and surface scattering. With this in mind, it is therefore clear that also the spread of values for the absorption cross-section peak of a single nanorod will be limited. Focusing the attention again on Figure S3a, it is visible that the absorption peak cannot go beyond $4 \cdot 10^{-15}$ m^2 for $AR = 3.5$, and beyond $5 \cdot 10^{-15}$ m^2 for $AR = 4$, even neglecting the plasmonic surface scattering. As already stated above, this threshold increases with increasing aspect ratio. It could be therefore assumed that, given the measured plasmonic linewidths, all the absorption peaks roughly $> 3 \cdot 10^{-15}$ m^2 for $\lambda_{SPR} < 800$ nm, and $> 3.5 \cdot 10^{-15}$ m^2 for $\lambda_{SPR} > 800$ nm, are due to aggregations of two or more nanorods, rather than a single one.

Supporting Section S3. Silicon nitride substrate

FEM simulations have been performed to study also how to the substrate affects the absorption cross-section spectrum for a individual gold nanorod. This analysis is carried out by evaluating the absorption cross-section for different thicknesses of the silicon nitride slab placed underneath the nano-absorber (see Figure 5b in the main text). For sake of simplic-

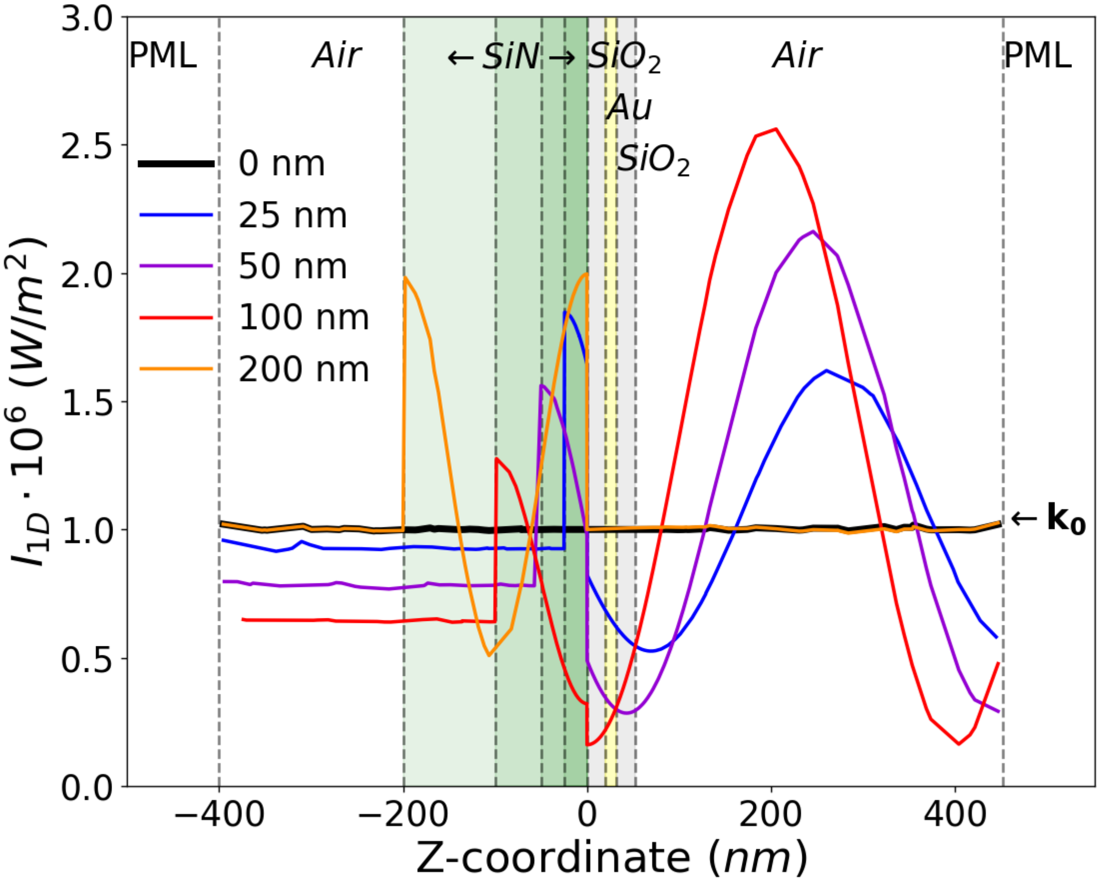

Figure S4: FEM simulated intensity distribution along a 1D cut line passing in the center of the physical domain at a wavelength of 800 nm, for different silicon nitride slab thicknesses. The intensities are the results to the FEM first step, where only the slab is simulated, without any gold nanorod on top of it. The vertical lines and the relative colors show the positions of each element along the cut line: white, air; grey, silica; yellow, gold; green of different intensities, the different silicon nitride slabs.

ity, the study is performed for one single wavelength (here 800 nm), but it can be extended to the whole spectrum considered in this work. Figure S4 shows the intensity distribution at 800 nm wavelength along a cut line parallel to the optical axis, passing in the center of the simulated physical domain. The intensities refer to the FEM first step, where only the silicon nitride slab is taken into account and the individual nanorod is not considered. Here, the full field formulation is used, i.e. an input electric field is defined on an input port on the simulated domain, and the total electric field, sum of the input and the refracted field, is calculated. In this way, it is possible to understand how the light interference due to the presence of the substrate affect ultimately the absorption cross-section of the nano-absorber. The vertical lines and the related colors, together with the tags, are plotted to indicated the positions occupied by each element along the central cut line in the physical domain. Even if not present in the simulation first step, the gold core and the silica coating of the nanorod are included for sake of clarity. The input electric field (indicated by $\mathbf{k_0}$ in the plot) enters the domain from the right-side (Z-coordinate positive values) and propagate towards the Z-coordinate negative values. Of particular interest is how the full field intensity shows different interference patterns for different silicon nitride slab thicknesses. Due to this effect, the intensity distribution in the proximity of the nanorod assumes different values for the different cases. For instance, going from 0 nm thickness (black curve) to 100 nm (red curve) the intensity in the nanorod's position decreases (yellowish region of z-values), resulting in a reduction of the absorption cross-section at 800 nm wavelength. For a thickness of 200 nm (dark orange curve), the silicon nitride slab acts as if it were transparent to this wavelength, resulting in a intensity distribution outside the slab almost identical to the case where no substrate is present at all. This is due to the value assumed by the refractive index n of the silicon nitride at this wavelength (the spectral distribution of the silicon nitride index of refraction has been taken from Ref. 16). For a vacuum wavelength of $\lambda_0 = 800$ nm, $n \approx 2$ inside the slab, which results in an effective wavelength $\lambda_n = \lambda_0/n \approx 400$ nm in the silicon nitride region, equivalent to half of the substrate thickness for the last curve in Figure S4

(dark orange). Therefore, half of the wavelength is contained in the slab, making it transparent to the incident electromagnetic wave. In this way, the absorption cross-section is going to be almost of the same magnitude as for the free space case. Indeed, the same electric field will enter the Equation (S1), resulting in equal electromagnetic energy losses. Consequently, a periodic pattern is recovered between the absorption cross-section at a specific wavelength and the thickness of the substrate, as it has been shown in Figure 5b in the main text.

Supporting Section S4. Signal-to-Noise ratio comparison

With the advantage of a reduction in experimental complexity compared to other label-free techniques, nanomechanical photothermal spectromicroscopy offers a highly sensitive approach to measure single particle and molecule. To show that, a comparison of the signal-to-noise ratio (SNR) among different label-free single-molecule technique is carried out (see the main text).[17] The three main quantity used to have a meaningful comparison are: the experimental SNR itself SNR_{exp}, as given by the authors (when not explicitly stated, averaged values are used instead); the power absorbed by the sample under study P_{heat}; the time required to perform a meaningful experiment τ_m. Table S1 summarizes all the useful information for the calculations developed in the main text. Each technique has been referenced with the corresponding work from which the experimental values have been extracted. Together with the three aforementioned quantities, Table S1 displays also the type of sample analyzed, as well as its corresponding absorption cross-section σ_{abs} and the pump intensity used to stimulated it (taking $1/e^2$ as definition of the laser beam waist). For sake of clarity, some of the techniques taken into account in Table S1 could performed only microscopy studies at the time of publication of the referenced works. Exception done for ground-state depletion microscopy, all the other techniques started to offer also spectroscopy capabilities in the last years.

Table S1: Parameters of the different techniques for the SNR comparison. SMS: spatial modulation spectroscopy; GSD: ground-state depletion microscopy; PCM: photothermal constrast microscopy; OMM: optical microresonator microscopy; NPM: nanomechanical photothermal microscopy; NPSM: nanomechanical photothermal spectromicroscopy. NG: not given. *: at the time of publication. **: assumed values.

**Technique**	**Capability***	**Pump Intensity** (kW/cm^2)	**Sample**	σ_{abs} (m^2)	P_{heat} (pW)	τ_m (ms) (avgs)	SNR_{exp}
UV-Vis Extinc.[18]	Spectro-microscopy	NG	Nanorod	NG	1274**	208**	6.38**
SMS[19]	Spectro-microscopy	22	Metal cluster	$4.3 \cdot 10^{-16}$	93620	10000*	2000
Extinc. + Bal.Det.[20]	Microscopy	280	TDI dye	$1.8 \cdot 10^{-19}$	508	2 (10x)	5.7
GSD[21]	Microscopy	590	Atto dye	$5 \cdot 10^{-20}$	294	30 (20x)	3.7
PCM (Glycerol)[22]	Microscopy	9300	BHQ	$4 \cdot 10^{-20}$	1000	300	10
PCM (5CB)[23]	Microscopy	28	Nanosphere	$4.8 \cdot 10^{-16}$	132000	20	78
PCM (Xe)[24]	Microscopy	28	Nanosphere	$4.8 \cdot 10^{-16}$	64	50	9.4
PCM (Xe)[25]	Microscopy	0.45	CP	$4 \cdot 10^{-18}$	64	30	10
OMM[26]	Spectro-microscopy	$2 \cdot 10^{-4}$	Nanorod	$1 \cdot 10^{-14}$	20	1000 (30x)	2
OMM[27]	Spectro-microscopy	522	CP	$8 \cdot 10^{-19}$	4100	100	4
NPM[28]	Microscopy	35.4	Atto dye	$4.8 \cdot 10^{-20}$	6.3	40	70
NPSM	Spectro-microscopy	4.98	Nanorod	$2.5 \cdot 10^{-15}$	120947	200	30759

Supporting Section S5. Relative power responsivity characterization

One of the main figures of merit of nanomechanical resonators for photothermal spectro-microscopy applications is its relative power responsivity R_P, which defines the amount of relative frequency shift experienced by the resonator upon absorption of 1 W of power (see main text Equation (3)). Here, it has been experimentally determined for the fundamental mechanical mode of different resonators as follows: the thermomechanical noise spectrum of the drumhead resonator has been measured with a Laser Doppler Vibrometer (MSA 500, Polytec GmbH) for different values of the probing laser power P_0 at 633 nm wavelength (Figure S5a). From these spectra, the resonance frequencies are extracted as a function of

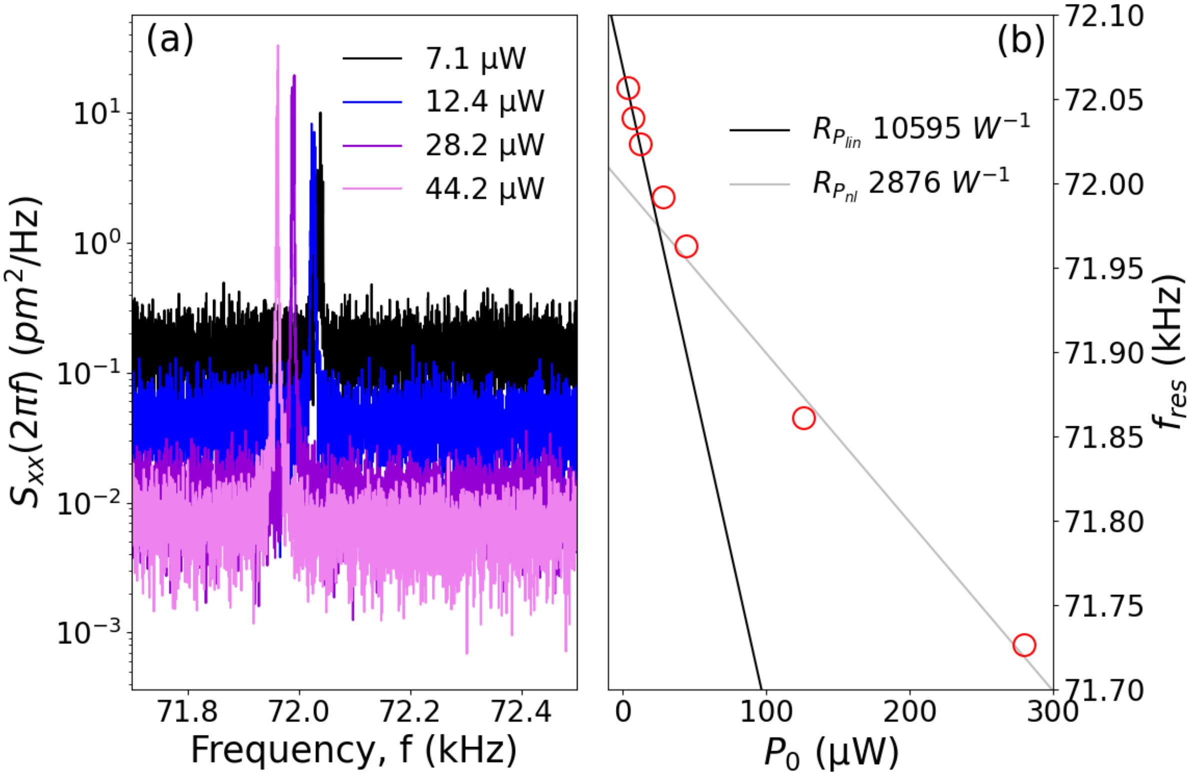

Figure S5: (a) Thermomechanical noise spectra for a drumhead resonator measured with a Laser Doppler Vibrometer, for four different probing laser power: 7.1, 12.4, 28.2, and 44.2 µW. (b) Mechanical resonance frequencies as a function of the input laser power P_0.

the input laser power P_0, and the data are fitted with a straight line (see Figure S5b, black curve)

$$f_{res}(P_0) = m \cdot P_0 + q. \tag{S4}$$

The y-intercept q corresponds to the heating-free resonance frequency $q = f_0$, while its gradient m corresponds to the product $m = \alpha_{abs}(\lambda) \cdot R_P \cdot f_0$. $\alpha_{abs}(\lambda)$ is the SiN slab wavelength-dependent absorption coefficient, whose value for a thickness of 50 nm at 633 nm wavelength is roughly equal to $\alpha_{abs}(633\ nm) = 0.5\%$.[16] For this specific nanoresonator, a relative power responsivity of $R_{P_{lin}} = 10595\ W^{-1}$ has been found.

It is also worth noting that a second, nonlinear power response regime is visible in Figure S5b (grey curve), showing a smaller slope ($R_{P_{nl}} = 2876\ W^{-1}$, one order of magnitude smaller than $R_{P_{lin}}$). This effect can be explained as a result of an increase of the thermal radiation emission from the SiN drumhead due to the resonator temperature T increase over the thermal bath temperature T_0, ultimately resulting in a higher resonator heat dissipation $q_{rad} = -\epsilon_{rad}\sigma_{SB}(T^4 - T_0^4)$.[29] ϵ_{rad} and σ_{SB} are the SiN emissivity and the Stefan-Boltzmann constant, respectively.

The power threshold that defines tha passage from the linear to the nonlinear regime is $P_{0_{threshold}} = 30\ \mu W$ ca. at 633 nm wavelength. For the range of wavelengths available with the Ti:Sapphire laser (720 - 900 nm), 50 nm thick SiN absorbes less, increasing the power threshold and allowing the full exploitation of the power responsivity of the drumhead resonator (see Figure S6). Finally, for the sake of clarity, it is interesting to mention that the different baselines characterizing the recorded thermomechanical noise spectra of Figure S5a reduce for higher vibrometer laser power, since the interferometric signal-to-noise ratio increases as the probing interferometric laser power P_0 increases.

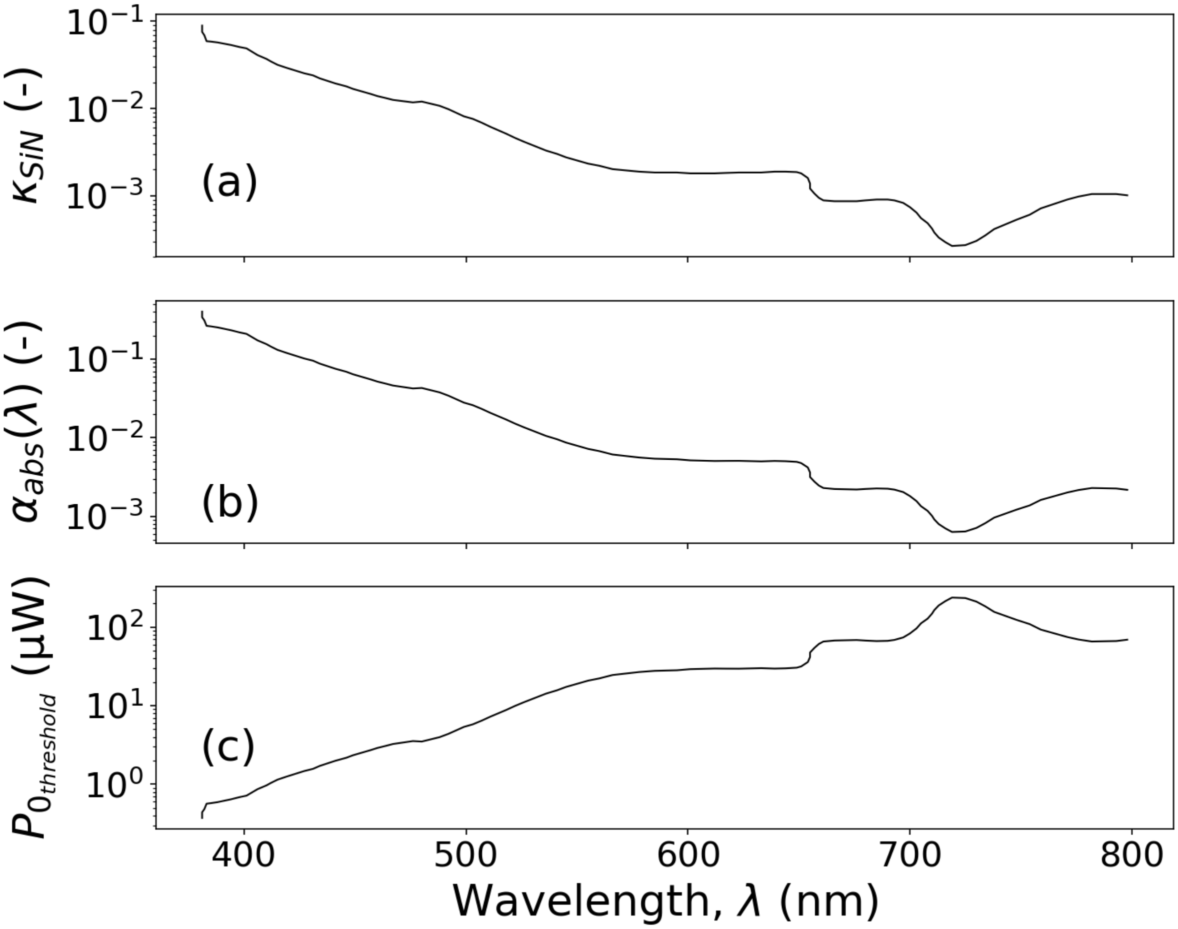

Figure S6: (a) Imaginary part of the complex refractive index $\tilde{n}$ for low-stress SiN.[30] (b) Estimated absorption coefficient from (a) assuming 50 nm thickness. (c): Calculated power threshold, assuming a linear relation with the absorption coefficient given in (b).

Supporting Section S6. Nanorod thermal time constant estimation.

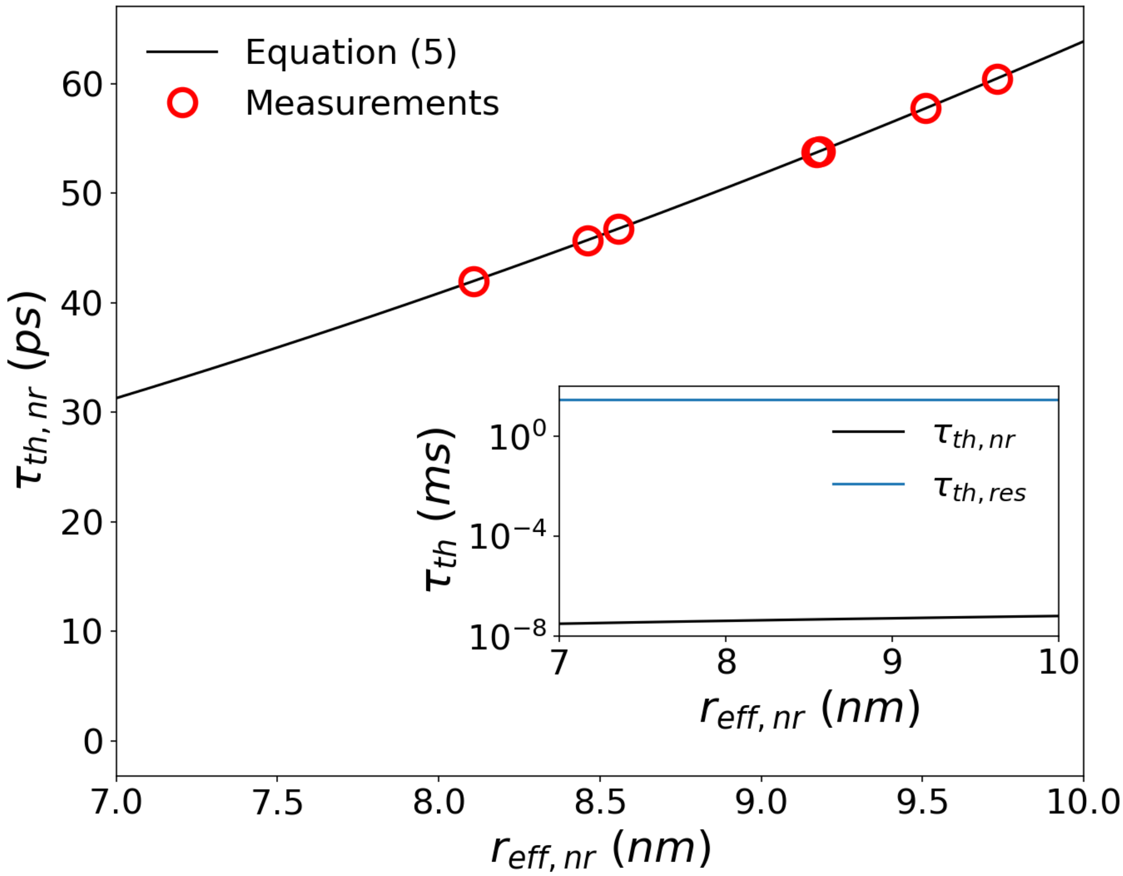

Figure S7: Estimated thermal time constant of the measured individual nanorods (red empty circles) as a function of the effective radius of a sphere of identical volume compared to the corresponding nanorod. (Inset) Comparison between the estimated nanorod thermal time constant and the measured mechanical resonator time constant.

From the extracted volumes of the individual nanorods (see main text), it possible to have an estimation of the time required for the nanorod to reach a steady-state temperature distribution upon illumination with a continuous-wave light source (see Equation (5) in the main text). Figure S7 show the calculation of the individual nanorods' thermal time constant

(red empty circle), which lies in the range 40-60 ps. In the inset, it is plotted the measured mechanical resonator thermal time constant $\tau_{th,res}$ for sake of completeness.

Supporting Section S7. SEM sample size distribution

The nanorod sample size distribution has been also measured with the help of scanning electron microscopy (SEM). Figure S8 shows the corresponding histograms for both the length and radial diameter.

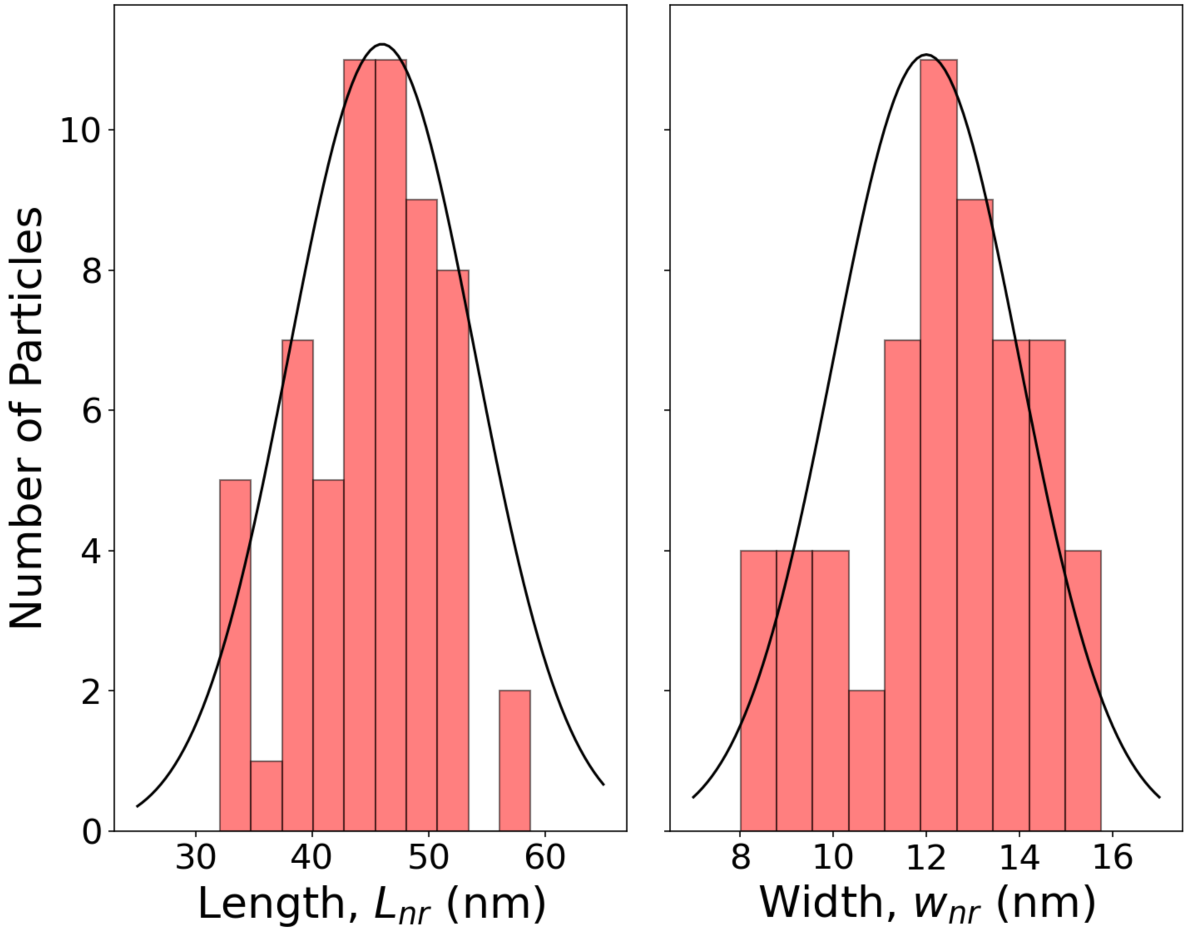

Figure S8: (a) Nanorod length L_{nr} distribution obtained from SEM images. (b) Nanorod radial diameter w_{nr} distribution obtained from SEM images.

References

(1) Lee, K. S.; El-Sayed, M. A. Dependence of the enhanced optical scattering efficiency relative to that of absorption for gold metal nanorods on aspect ratio, size, end-cap shape, and medium refractive index. *J. Phys. Chem. B* **2005**, *109*, 20331–20338.

(2) Muskens, O. L.; Bachelier, G.; Fatti, N. D.; Vallée, F.; Brioude, A.; Jiang, X.; Pileni, M. P. Quantitative absorption spectroscopy of a single gold nanorod. *J. Phys. Chem. C* **2008**, *112*, 8917–8921.

(3) Davletshin, Y. R.; Lombardi, A.; Cardinal, M. F.; Juvé, V.; Crut, A.; Maioli, P.; Liz-Marzán, L. M.; Vallée, F.; Fatti, N. D.; Kumaradas, J. C. A quantitative study of the environmental effects on the optical response of gold nanorods. *ACS Nano* **2012**, *6*, 8183–8193.

(4) Tian, L.; Chen, E.; Gandra, N.; Abbas, A.; Singamaneni, S. Gold nanorods as plasmonic nanotransducers: Distance-dependent refractive index sensitivity. *Langmuir* **2012**, *28*, 17435–17442.

(5) Johnson, P.; Christy, R. Optical-Constants Of Noble-Metals Phys. Rev. B 6(12), 4370–4379 (1972). *Phys. Rev. B* **1972**, *1318*, 4370–4379.

(6) Knight, M. W.; Wu, Y.; Lassiter, J. B.; Nordlander, P.; Halas, N. J. Substrates matter: influence of an adjacent dielectric on an individual plasmonic nanoparticle. *Nano Lett.* **2009**, *9*, 2188–2192.

(7) COMSOL Inc., Scatterer on Substrate. `https://www.comsol.com/model/scatterer-on-substrate-14699`, (accessed 2023-09-06).

(8) Grand, J.; Ru, E. C. L. Practical Implementation of Accurate Finite-Element Calculations for Electromagnetic Scattering by Nanoparticles. *Plasmonics* **2020**, *15*, 109–121.

(9) Nima, Z. A.; Davletshin, Y. R.; Watanabe, F.; Alghazali, K. M.; Kumaradas, J. C.; Biris, A. S. Bimetallic gold core-silver shell nanorod performance for surface enhanced Raman spectroscopy. *RSC Adv.* **2017**, *7*, 53164–53171.

(10) Novo, C.; Gomez, D.; Perez-Juste, J.; Zhang, Z.; Petrova, H.; Reismann, M.; Mulvaney, P.; Hartland, G. V. Contributions from radiation damping and surface scattering to the linewidth of the longitudinal plasmon band of gold nanorods: A single particle study. *Phys. Chem. Chem. Phys.* **2006**, *8*, 3540–3546.

(11) Juvé, V.; Cardinal, M. F.; Lombardi, A.; Crut, A.; Maioli, P.; Pérez-Juste, J.; Liz-Marzán, L. M.; Fatti, N. D.; Vallée, F. Size-dependent surface plasmon resonance broadening in nonspherical nanoparticles: Single gold nanorods. *Nano Lett.* **2013**, *13*, 2234–2240.

(12) Li, Z.; Mao, W.; Devadas, M. S.; Hartland, G. V. Absorption Spectroscopy of Single Optically Trapped Gold Nanorods. *Nano Lett.* **2015**, *15*, 7731–7735.

(13) Germer, T. A. pySCATMECH : A Python Interface to the SCATMECH Library. `https://pages.nist.gov/pySCATMECH/index.html`, (accessed 2023-09-06).

(14) Lioi, D. B.; Izor, S. N.; Varshney, V.; DeJarnette, H. M.; Derosa, P. A.; Kennedy, W. J. Effective medium approximation for the dielectric environment of coated gold nanorods. *Opt. Mater. Express* **2022**, *12*, 3577.

(15) Brioude, A.; Jiang, X. C.; Pileni, M. P. Optical properties of gold nanorods: DDA simulations supported by experiments. *J. Phys. Chem. B* **2005**, *109*, 13138–13142.

(16) Philipp, H. R. Optical Properties of Silicon Nitride. *J. Electrochem. Soc.* **1973**, *120*, 295.

(17) Goldsmith, R., Optical Microresonators as Single-Molecule Spectrometers.

https://home.uni-leipzig.de/~physik/sites/photothermal-webinar/randall-goldsmith/, (accessed 2023-07-15).

(18) Ming, T.; Zhao, L.; Yang, Z.; Chen, H.; Sun, L.; Wang, J.; Yan, C. Strong polarization dependence of plasmon-enhanced fluorescence on single gold nanorods. *Nano Lett.* **2009**, *9*, 3896–3903.

(19) Arbouet, A.; Christofilos, D.; Fatti, N. D.; Vallée, F.; Huntzinger, J. R.; Arnaud, L.; Billaud, P.; Broyer, M. Direct measurement of the single-metal-cluster optical absorption. *Phys. Rev. Lett.* **2004**, *93*, 127401.

(20) Celebrano, M.; Kukura, P.; Renn, A.; Sandoghdar, V. Single-molecule imaging by optical absorption. *Nat. Photon.* **2011**, *5*, 95–98.

(21) Chong, S.; Min, W.; Xie, X. S. Ground-state depletion microscopy: Detection sensitivity of single-molecule optical absorption at room temperature. *J. Phys. Chem. Lett.* **2010**, *1*, 3316–3322.

(22) Gaiduk, A.; Yorulmaz, M.; Ruijgrok, P.; Orrit, M. Room-Temperature Detection of a Single Molecule's Absorption by Photothermal Contrast. *Science* **2010**, *330*, 353–356.

(23) Chang, W. S.; Link, S. Enhancing the sensitivity of single-particle photothermal imaging with thermotropic liquid crystals. *J. Phys. Chem. Lett.* **2012**, *3*, 1393–1399.

(24) Ding, T. X.; Hou, L.; Meer, H. V. D.; Alivisatos, A. P.; Orrit, M. Hundreds-fold Sensitivity Enhancement of Photothermal Microscopy in Near-Critical Xenon. *J. Phys. Chem. Lett.* **2016**, *7*, 2524–2529.

(25) Hou, L.; Adhikari, S.; Tian, Y.; Scheblykin, I. G.; Orrit, M. Absorption and Quantum Yield of Single Conjugated Polymer Poly[2-methoxy-5-(2-ethylhexyloxy)-1,4-phenylenevinylene] (MEH-PPV) Molecules. *Nano Lett.* **2017**, *17*, 1575–1581.

(26) Heylman, K. D.; Thakkar, N.; Horak, E. H.; Quillin, S. C.; Cherqui, C.; Knapper, K. A.; Masiello, D. J.; Goldsmith, R. H. Optical microresonators as single-particle absorption spectrometers. *Nat. Photon.* **2016**, *10*, 788–795.

(27) Horak, E. H.; Rea, M. T.; Heylman, K. D.; Gelbwaser-Klimovsky, D.; Saikin, S. K.; Thompson, B. J.; Kohler, D. D.; Knapper, K. A.; Wei, W.; Pan, F.; Gopalan, P.; Wright, J. C.; Aspuru-Guzik, A.; Goldsmith, R. H. Exploring Electronic Structure and Order in Polymers via Single-Particle Microresonator Spectroscopy. *Nano Lett.* **2018**, *18*, 1600–1607.

(28) Chien, M. H.; Brameshuber, M.; Rossboth, B. K.; Schütz, G. J.; Schmid, S. Single-molecule optical absorption imaging by nanomechanical photothermal sensing. *PNAS* **2018**, *115*, 11150–11155.

(29) Piller, M.; Sadeghi, P.; West, R. G.; Luhmann, N.; Martini, P.; Hansen, O.; Schmid, S. Thermal radiation dominated heat transfer in nanomechanical silicon nitride drum resonators. *Appl. Phys. Lett.* **2020**, *117*, 034101.

(30) Poenar, D. P.; Wolffenbuttel, R. F. Optical properties of thin-film silicon-compatible materials. *Appl. Opt.* **1997**, *36*, 5122–5128.
